# Aptamer‐Mediated Artificial Synapses for Neuromorphic Modulation of Inflammatory Signaling via Organic Electrochemical Transistor

**DOI:** 10.1002/advs.202509545

**Published:** 2025-08-04

**Authors:** Yuqing Ding, You Kuai, Rongpei Li, Xinzhao Xu, Bo Wang, Zhihui Wang, Yanfang Liu, Yuchao Dong, Shunjie Chen, Meng Guo, Yunqi Liu, Yan Zhao

**Affiliations:** ^1^ Department of Materials Science Fudan University Shanghai 200433 P. R. China; ^2^ Department of Nephrology Shanghai Fourth People's Hospital School of Medicine Tongji University Shanghai 200434 P. R. China; ^3^ Department of Respiratory and Critical Care Medicine Changhai Hospital Naval Medical University Shanghai 200433 P. R. China; ^4^ National Key Laboratory of Medical Immunology &Institute of Immunology Navy Medical University Shanghai 200433 P. R. China

**Keywords:** aptamer‐based biosensor, interleukin‐6 sensing, neuromorphic interface, organic electrochemical transistor, synaptic plasticity

## Abstract

Artificial synaptic devices that mimic neuromorphic signal processing hold great promise for bioelectronic interfaces. However, most systems remain limited to physical stimuli or electroactive small molecules, lacking the ability to transduce biologically relevant protein signals. To address this limitation, an aptamer‐mediated aqueous artificial synaptic transistor is developed capable of selectively responding to the interleukin‐6 (IL‐6) signal, a specifically expressed protein of inflammatory stress, via gate‐voltage‐induced synaptic modulation in biologically relevant electrolyte environments. Guided by molecular docking simulations, high‐affinity aptamer sequences are identified for robust recognition of IL‐6. The device demonstrates precise IL‐6 capture and translation into neuromorphic electrical signals across various biological electrolytes (PBS, albumin, serum), with linear detection from 0.5 pm to 50 nm. Moreover, the device can convert IL‐6 binding events into time and concentration‐dependent electrical outputs, exhibiting significant synaptic plasticity and memory retention. When implanted into the caudal vein of sepsis mice, the device stably monitors IL‐6 level and maintains reliable synaptic response to inflammatory‐triggered elevations. Machine learning analysis enables accurate discrimination between normal and pathological states from device‐generated signals. By bridging biochemical signals with neuromorphic encoding, this system outlines a conceptual framework for future integration between artificial and biological neural units, contributing to the hybrid neurosensory systems.

## Graphical abstract

An aptamer‐mediated neuromorphic interface based on organic electrochemical transistors selectively transduces interleukin‐6 (IL‐6) signals into neuromorphic electrical outputs. This protein‐driven neuromorphic plasticity, responsive in physiological and in vivo environmental electrolyte, bridges biochemical signaling with artificial neural encoding, offering a molecularly tunable platform for biochemical signal encoding and bridging artificial neuro‐signals with biological communication pathways.

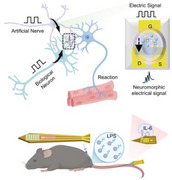

## 1 Introduction

Bioinspired neuromorphic electronics are rapidly developing to process and transduce physiological signals, offering promising routes for biomedical interfaces, prosthetics, and human‐machine.^[^ ^1^, ^2^, ^3^, ^4^ ^]^ Artificial synaptic transistors, which emulate key dynamic features of biological synapses such as plasticity and memory retention,^[^ ^5^, ^6^ ^]^ are regarded as ideal candidates for next‐generation bioelectronic interfaces. Their potential to generate adaptive, cumulative, and time‐dependent signals makes them fundamentally compatible with the information processing modes of biological neural networks.^[^ ^7^ ^]^ Most artificial synapses rely on physical stimuli such as light or voltage and cannot sense biochemical cues that mediate real cellular communication. This limits their compatibility with biological signaling environments. To enable more realistic modulation, neuromorphic systems must incorporate chemical communication and biomolecule‐mediated plasticity. Equipping artificial synapses with the ability to interpret molecular signals and encode them into dynamic electrical responses could bridge artificial and biological circuits, laying the foundation for hybrid networks that enhance physiological perception through embedded neuromorphic signaling.

While previous efforts have focused on redox‐active small molecules,^[^ ^8^, ^9^ ^]^ biological signaling is predominantly governed by proteins, especially cytokines, that orchestrate neural, immune, and metabolic communication.^[^ ^10^, ^11^ ^]^ Realizing protein‐mediated neuromorphic modulation is thus essential for embedding artificial neuro signals into biological signaling pathways in a functionally meaningful way. However, the inherent non‐electroactivity and environmental sensitivity of proteins pose significant challenges for their effective signal transduction between the interface of biological neurons and artificial synaptic devices.

To solve the problems faced in protein molecular signal regulation, aptamers were introduced as molecular recognition elements in this study. Aptamers, as a kind of nucleic acid sequence, can specifically bind target molecules by their flexible design, high chemical stability, and environmental adaptability, and achieve electrical signal conversion through conformational changes, thus overcoming the shortcomings of complex and easy inactivation of antibody preparation and environmental sensitivity of enzymes.^[^ ^12^ ^]^ As a representative model, we focus on interleukin‐6 (IL‐6), a multifunctional cytokine that acts as a key driver of inflammation and the subsequent tissue damage.^[^ ^13^, ^14^, ^15^ ^]^IL‐6 plays a key role in immune regulation and is rapidly upregulated during infection, injury, and cytokine storms. Its concentration‐dependent dynamics reflect systemic physiological states. Converting such cues into neuromorphic signals enables artificial encoding of immune activity and offers a signal format compatible with biological neural processing, supporting future integration for physiological state interpretation and sensory modulation.

To realize protein‐driven neuromorphic signal modulation, we construct an aptamer‐mediated aqueous‐gated artificial synaptic transistor (AAAST) that translates biochemical recognition into adaptive electronic behavior. We adopt an organic electrochemical transistor (OECT) architecture, particularly based on biocompatible poly(3,4‐ethylenedioxythiophene): poly(styrene sulfonate) (PEDOT: PSS), to facilitate efficient ion‐electron coupling at the bioelectronic interface. This platform not only emulates biological synaptic plasticity but also enables the encoding of biochemical cues into dynamic neuromorphic outputs, laying the foundation for hybrid information transmission and enhanced physiological perception. The device operates reliably in physiologically relevant environments, including phosphate buffered saline (PBS), albumin, and serum, exhibiting high sensitivity across an IL‐6 concentration range of 0.5 pm to 50 nm. It also realizes IL‐6‐induced time and concentration‐dependent conductance modulation, including cumulative memory effects, frequency filtering, and paired‐pulse facilitation. Furthermore, leveraging machine learning enables the interpretation and classification of these neuromorphic signals, allowing the device to distinguish between normal and pathological biochemical states, thereby creating a structured framework for biochemical signal encoding and interpretation. When implanted into mice, the device remains stable and exhibits an instantaneous response to inflammatory conditions, confirming its compatibility and interaction with the biological system. This work provides a step toward implantable neuromorphic interfaces for intelligent health monitoring, hybrid neurosensory systems, and neuro‐prosthetics.

## 2 Results and Discussion

### 2.1 Design and Characterization of the Aptamer‐Functionalized Device

**Figure 1:**
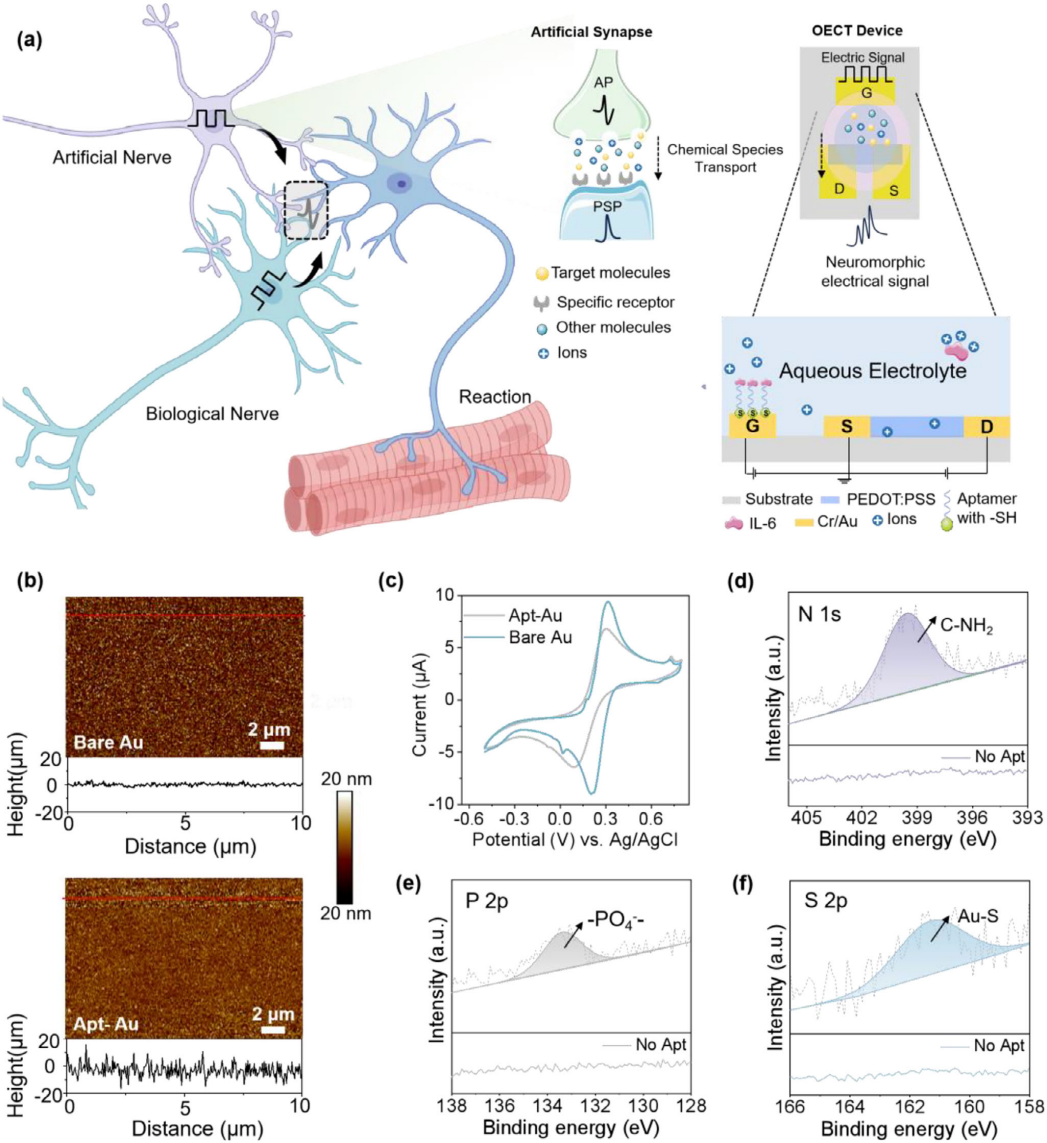
Integration of artificial synapses with biological signaling. a) Conceptual and structural illustration of AAAST, enabling signal coupling between artificial and biological neural systems. The system mimics biological synaptic function by applying presynaptic stimulation at a DNA aptamer‐functionalized gate electrode and generating postsynaptic responses at the semiconductive PEDOT: PSS channel, highlighting the potential for bidirectional communication and feedback integration across bioelectronic interfaces. Characterization of functionalized gate electrodes. b)Atom force microscope (AFM) images of bare Au gate with (top) and without DNA (bottom) aptamer modification. c) The cyclic voltammetry (CV) curve of the Bare Au and Apt‐Au. X‐ray photoelectron spectroscopy (XPS) spectra of d) N 1s, e) P 2p, and f) S 2p with/without the DNA aptamer modification.

A conceptual illustration of a protein‐responsive artificial synaptic transistor bridging molecular inputs and neuromorphic outputs is depicted in **Figure** **1**a (left). Upon recognizing biomolecules, the device generates time‐dependent conductance modulation resembling synaptic plasticity. The schematic envisions the integration of such artificial neurosignals alongside biological neural pathways, where both signal streams converge to influence downstream responses. This hybrid interface introduces a new signal modulation route via molecular recognition, offering the potential for bioelectronic augmentation of neural signaling and enhanced physiological perception.

AAAST is designed with a three‐terminal configuration in Figure 1a (right), consisting of a gate electrode, an organic semiconducting channel, and source/drain to capture neuromorphic signal dynamics. The organic channel was formed by spin‐coating PEDOT: PSS between the source and drain electrodes, enabling ion‐electron coupling for effective current modulation. In this framework, the gate input serves as an analog of the presynaptic action potential (AP), while the modulated channel current reflects the postsynaptic potential (PSP), enabling a biologically inspired representation of signal transmission and synaptic plasticity. The gate electrode was functionalized with thiolated DNA aptamers specific to IL‐6 via self‐assembled Au‐S bonding, enabling selective recognition of this non‐electroactive protein molecule.^[^ ^16^ ^]^ To minimize nonspecific adsorption, unbound sites were passivated using 6‐mercapto‐1‐hexanol (MCH).^[^ ^17^, ^18^ ^]^ PDMS well confined the electrolyte environment, including PBS, albumin, or human serum, allowing device operation under physiologically relevant conditions. Upon binding IL‐6, the DNA aptamer undergoes a conformational change that modulates the electric double layer (EDL) at the gate/electrolyte interface, dynamically altering the channel current and translating biomolecular recognition events into neuromorphic electrical responses.^[^ ^19^ ^]^ Such a mechanism supports a biomolecular‐to‐neuromorphic transduction strategy, potentially contributing to molecular‐level signal encoding in bioelectronic interfaces.^[^ ^20^ ^]^ The fabrication procedures are detailed in the Experimental Section.

We characterized the surface morphology and roughness of the aptamer‐modified gold layer using atomic force microscopy (AFM). As shown in Figure 1b, the surface roughness of the gate electrode increased significantly, from 2.5 nm for the bare gold electrode (top) to 5.9 nm after aptamer modification (bottom). This increase in roughness provides clear evidence of successful aptamer functionalization on the gold gate surface. Further confirmation is provided by the height map included at the bottom of each figure, which demonstrates the enhanced surface roughness modification and the effective immobilization of the DNA‐aptamer. Besides, the similar trend of the surface morphology before and after the aptamer modification has also been characterized by scanning electron microscopy (SEM), as shown in Figure S1 (Supporting Information). Kelvin Probe Force Microscopy (KPFM) was used to measure the contact potential difference (*V*_CPD_) of the gold electrode, which increased from 101 mV (*V*_Au_) to 280 mV (*V*_Apt_) after functional layer assembly in Figure S2 (Supporting information). This indicates a reduced work function due to insulating layers that lower electron injection efficiency.^[^ ^21^ ^]^

The electrochemical properties of the gold electrode before and after aptamer modification were evaluated using cyclic voltammetry (CV) and electrochemical impedance spectroscopy (EIS) in a PBS solution containing 5 mmol L^−1^ of Fe[(CN)₆]^3^−/Fe[(CN)₆]⁴−. The CV results in Figure 1c show a marked decrease in electrochemical activity upon aptamer modification. This reduction is attributed to the insulating properties of the DNA‐aptamer, which forms a protective layer on the electrode surface, reducing electrochemical activity.^[^ ^22^ ^]^ The EIS measurements also exhibit similar results, as shown in Figure S3 (Supporting information). The Nyquist plot of the bare gold electrode reveals a small semicircle in the impedance spectrum, reflecting its excellent conductivity due to the inherent properties of gold. Upon modification with the DNA‐aptamer, the semicircle in the Nyquist plot broadens significantly toward larger Z’ values, signifying an increase in charge transfer resistance (R_ct_). This increase occurs because the aptamer layer acts as a barrier to negatively charged redox species, impeding their access to the electrode surface. These findings are consistent with the reduction in electrochemical activity observed in the CV measurements, further validating the successful functionalization of the gold electrode with the DNA‐aptamer.

Figure 1d–f shows the high‐resolution XPS spectra of N 1s, P 2p, and S 2p for bare gold (Au) and aptamer‐modified gold (Apt‐Au) surfaces. The appearance of a distinct N 1s peak (C‐NH_2_), a P 2p peak (‐PO_4_ bonds), and a characteristic S 2p peak (S‐Au bond) on the apt‐Au surface indicates the presence of nitrogen‐, phosphorus‐,^[^ ^23^ ^]^ and sulfur‐containing groups^[^ ^24^, ^25^ ^]^ from the DNA structure. Additional evidence comes from the high‐resolution C 1s spectra (Figure S4, Supporting information), which show components corresponding to C‐NH_2_ (287.6 eV), C‐N (286.1 eV), and C‐C (284.8 eV), consistent with the aptamer's chemical composition.^[^ ^26^, ^27^ ^]^ These XPS results collectively confirm the successful functionalization of the gold surface with the DNA‐aptamer.

### 2.2 Conversion of Biological Signals to Electrical Signals

**Figure 2:**
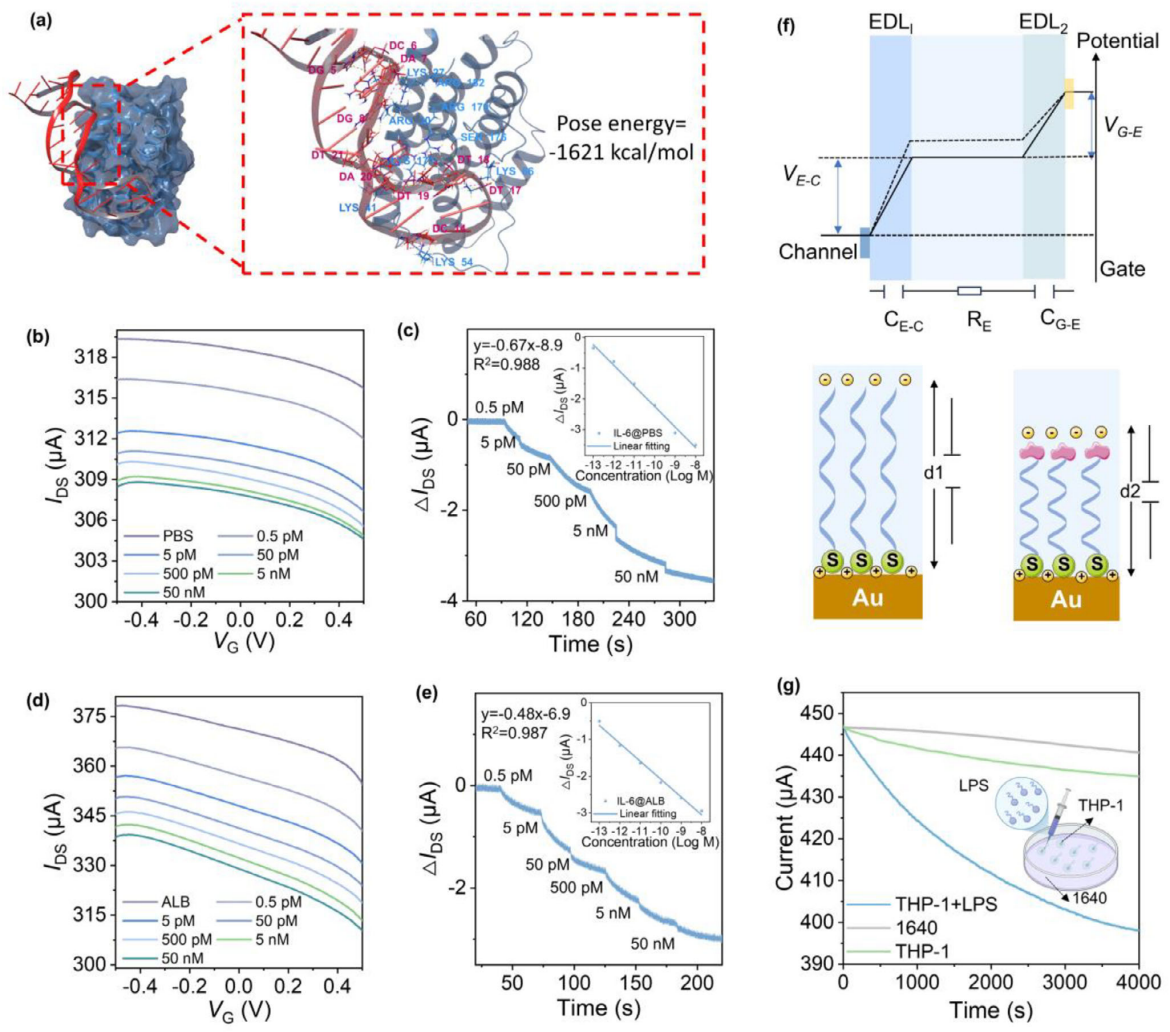
Detection of IL‐6 and sensing mechanism of the device for bio‐electric signal transduction a)Molecular docking of IL‐6 and DNA aptamer binding sites and interactions. b) Transfer characteristic curve of the device at different IL‐6 concentrations in PBS solution. c) Real‐time channel current response and the linear fit of Δ*I*_DS_ of the device at different IL‐6 concentrations in PBS solution. d) Transfer characteristic curves of the device at the different IL‐6 concentrations in ALB solution. e) Real‐time channel current response and the linear fit of Δ*I*_DS_ of the device at different IL‐6 concentrations in ALB solution. f) The potential distribution across the two EDLs of the device(top) and schematic diagram of charge and capacitance variation before and after the capture of IL‐6 protein (bottom). g) Real‐time current response for 1640, THP‐1, and THP‐1+LPS.

The DNA aptamer functions as a critical biorecognition element in the system to ensure selective recognition and signal transduction of the target molecule IL‐6. We first employed molecular docking to evaluate the interaction between IL‐6 and the aptamer, establishing a model of their binding interface, which revealed that the DNA aptamer binds tightly to IL‐6, with the top‐ranked pose exhibiting a PIPER energy of −1621 kcal mol^−1^ in **Figure** **2**a. Multiple hydrogen bonds, salt bridges, and π‐cation interactions were identified at the binding interface, indicating strong and specific affinity.^[^ ^28^ ^]^ Surface plasmon resonance (SPR) analysis was performed in Figure S5 (Supporting information), using a Biacore system with a CM5 chip to evaluate the binding affinity between IL‐6 protein and its aptamer. The results show a high‐affinity interaction, with an association rate constant (*K*_a_) of 2.21 × 10^3^ 1/(Ms), a dissociation rate constant (*K*_d_) of 6.52 × 10^−4^ 1/s, and a dissociation constant (*K*_D_) of 2.95 × 10^−7^ m, indicating effective and specific binding. Building on the strong binding affinity predicted by molecular docking between IL‐6 and the aptamer, we evaluated the device's biochemical recognition capability in solution conditions. This can validate the efficiency and specificity of molecular input capture within the AAAST, which serves as the molecular recognition stage of the neuromorphic signaling process. A PDMS well is used to confine electrolyte solutions, such as PBS and ALB. The device's response to varying IL‐6 concentrations in PBS was investigated by sweeping the gate voltage (*V*_G_) from −0.5 to 0.5 V while maintaining a constant source‐drain voltage (*V*_DS_) of 0.1 V. The transfer characteristics in Figure 2b reveal a clear trend that the source‐drain current (*I*_DS_) decreases monotonically with increasing IL‐6 concentration. This behavior, driven by the IL‐6 aptamer functionalized on the gate electrode, demonstrates the device's ability to transduce the biological signal of IL‐6 into a corresponding electrical response. The biosensor shows high sensitivity, detecting IL‐6 concentrations from 0.5 to 50 nm, which aligns with clinical ranges relevant to cytokine release syndrome.^[^ ^29^ ^]^ The real‐time response for IL‐6 detection, shown in Figure 2c, indicates a response time of just a few minutes across the tested concentration range. As IL‐6 concentration increases, the channel current decreases. At 0.5 pm, a detectable current change is observed, confirming the sensor's excellent detection threshold. The change in drain current (Δ*I*_DS_) and the concentration of IL‐6 can be fitted by the linear equation y = −0.67 × −8.9, where X = log C, and C is the concentration of IL‐6. The linear coefficient of determination R^2^ reaches 0.988, indicating a strong linear relationship between Δ*I*_DS_ and the concentration of IL‐6 in the range of 10^−13^ to 10^−8^ m. To ensure optimal surface functionalization for electrical response, we further evaluate aptamer concentrations of 0.2, 0.5, and 1 µm. Among these, 0.5 µm demonstrates the most stable and reliable real‐time electrical response and was therefore selected for all subsequent device experiments. The corresponding changes in drain current under IL‐6 stimulation for each aptamer concentration have been quantitatively analyzed and summarized in Figure S6 (Supporting Information). This comparison reveals that 0.2 or 1 µm resulted in relatively weak responses due to limited surface coverage or steric effects, or nonspecific interactions at too low or too high surface densities. In contrast, 0.5 µm achieved a well‐balanced interface, enabling both high sensitivity and signal stability.

To investigate the role of the DNA aptamer in signal transduction, we performed real‐time experiments with a device without the DNA aptamer modification on its side gate (Figure S7, Supporting Information). In these experiments, with increasing concentrations of IL‐6, the *I*_DS_ exhibited negligible changes. This result demonstrates that without the DNA aptamer, the device is unable to convert the IL‐6 biosignal into an electrical signal. Besides, the selectivity of the device is verified by adding non‐relevant biomolecules, such as urea, LA, and fructose (Fru), to the device (Figure S8, Supporting Information). In addition, control experiments were performed with structurally or functionally related cytokines, including interferon‐ γ (IFN‐ γ) and IL‐11. Similar to the results observed with unrelated small molecules, these cytokines failed to produce noticeable changes in current (Figure S9, Supporting Information). Specifically, we included the results of mixed‐cytokine experiments in which IL‐6 is combined with IL‐11 and IFN‐γ. The electrical response of the device to this cytokine mixture is nearly identical to that induced by IL‐6 alone, indicating that the presence of IL‐11 and IFN‐γ did not interfere with the specific recognition of IL‐6 by the aptamer, as shown in Figure S10 (Supporting Information). These results confirm the high molecular specificity and strong anti‐interference capability of the device in complex inflammatory environments. Therefore, these findings confirm that the device exhibits high selectivity for IL‐6, which is enabled by the specific recognition capability of the DNA aptamer functionalized on the gate electrode.^[^ ^30^ ^]^

To further evaluate performance in a more biologically complex environment, similar experiments were conducted in ALB solution. Despite the complex medium, the device retained high sensitivity and selectivity. The transfer curves in Figure 2d show the decrease of the current with increasing IL‐6 concentrations, and real‐time response curves in Figure 2e show comparable rapid signal modulation. The fitted linear relationship in ALB (y = −0.48x − 6.9, R^2^ = 0.987) confirms that the device maintains reliable IL‐6 responsiveness in physiological environments. Notably, the tested IL‐6 concentration range (0.5 pm–50 nm) spans those associated with chronic or acute inflammatory responses, highlighting the broad applicability of the device for real‐time monitoring of inflammatory states.^[^ ^31^ ^]^

The observed trend of decreasing *I*_DS_ with increasing IL‐6 concentrations can be explained using the electric double layer (EDL) model, as illustrated in Figure 2f. According to the Bernards model.^[^ ^32^ ^]^ The recognition and binding of IL‐6 occur at the gate electrode surface functionalized with DNA aptamers. As a negatively charged biomolecule, IL‐6 significantly increases the surface charge density upon binding, leading to the local enrichment of cations in the adjacent electrolyte to maintain charge neutrality.^[^ ^33^ ^]^This accumulation of positive ions near the gate elevates the local electrolyte potential relative to the gate, reducing the gate‐electrolyte potential difference (*V*_G–E_). Given that the total gate voltage (*V*_G_) applied in an OECT is distributed across the gate‐electrolyte and electrolyte‐channel interfaces according to *V*_G_ = *V*_G–E_+ *V*_E–C_, a reduction in *V*_G–E_ necessarily results in an increase in the *V*_E–C_ under constant *V*_G_. The elevated *V*_E–C_ enhances the migration of cations into the PEDOT: PSS channel, promoting de‐doping and converting the conductive PEDOT⁺ to its neutral, non‐conductive PEDOT⁰ form.^[^ ^34^ ^]^ Consequently, the channel conductivity decreases, leading to a reduction in the drain current. These changes are illustrated in the EDL model in Figure 2f (bottom), where the dashed lines indicate the altered potential profile following IL‐6 binding. The binding of IL‐6 induces a conformational change in the aptamer, causing it to fold and draw negative charges closer to the gate interface. This effectively reduces the spatial distance between the electrolyte and the gate electrode (from d1 to d2), which is equivalent to decreasing the dielectric thickness of the interfacial electrical double layer, increasing the gate‐electrolyte capacitance (C_G–E_), as shown in Figure S11 (Supporting Information). According to the voltage division rule across capacitors, this increase in C_G–E_ causes a decrease in *V*_G–E_ and a corresponding increase in *V*_E–C_, consistent with the trend observed in the channel current. To further validate the device's ability to detect biologically secreted IL‐6, we conducted cell‐based experiments using human‐derived macrophage‐like THP‐1 cells. Cells were cultured in the indicated medium and divided into two groups: a control group (unstimulated THP‐1 cells) and an experimental group treated with lipopolysaccharide (LPS, 5 µg/mL), a bacterial component commonly used to induce IL‐6 production in macrophages.^[^ ^35^ ^]^ Optical microscopy showed that control cells maintained a typical round and transparent morphology without pseudopodia, indicative of a resting, non‐activated state, as shown in Figure S12a (Supporting information). As shown in Figure 2g, the real‐time current response of the device was monitored after exposure to the respective culture medium. In the control group containing only THP‐1 cells and culture medium, the current remained relatively stable over time, indicating negligible IL‐6 secretion under basal conditions. In contrast, the THP‐1 + LPS group induced a pronounced decrease in channel current, following the previously established trend in which higher IL‐6 concentrations correspond to lower current levels. This result indicates sustained IL‐6 secretion by LPS‐stimulated macrophages and is consistent with the ELISA measurements shown in Figure S12b (Supporting Information). These results confirm the device's ability to detect cytokines secreted by immune cells in complex biological environments.

### 2.3 IL‐6‐Diven Neuromorphic Signal Modulation

**Figure 3:**
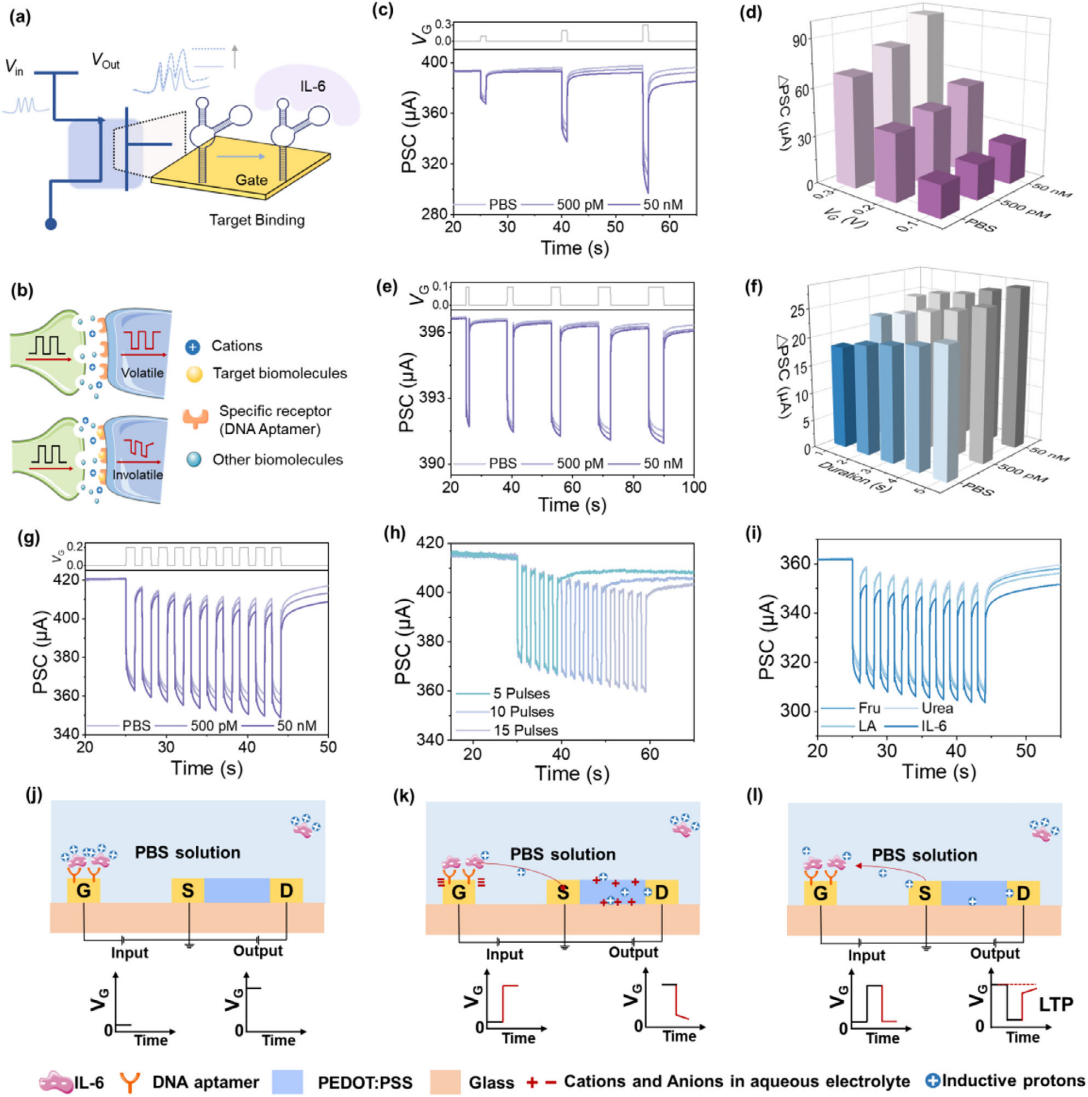
The neuromorphic functions of the AAAST device. a)The OECT configuration with a functionalized gate electrode, where IL‐6 binds to immobilized aptamers. b) Schematic of a biological synapse with selective receptors on the postsynaptic membrane. Non‐relevant biomolecules fail to open ion channels (top), while specific binding triggers channel opening and induces long‐term modulation (bottom). c) Short‐term and long‐term modulation of the postsynaptic channel current stimulated by a single voltage pulse (*V*_G_ = 0.1, 0.2, and 0.3 V, t  =  1s) at *V*_DS_ = 0.1 V in PBS solution. d) The ΔPSC for different pulse amplitudes. e)Synaptic behaviors of the device as a function of the pulse duration in PBS solution. f) The ΔPSC for a different duration. g) Cumulative pulse stimulations for the device. h) The cumulative memory effect of the device under 5, 10, and 15 successive pulses in the presence of IL‐6. i) Cumulative pulse stimulations of the same device in PBS solution containing different biomolecules, urea, LA, Fru, and IL‐6 (all in 500 pm). Schematic illustration of the working principle of the device with IL‐6 when j) no voltage pulse is applied, k)the channel current is induced by the applied voltage pulse, and l) when the pulse is removed.

Building on the demonstrated capability of the AAAST device to efficiently transduce molecular recognition into electrical signals, we next applied it to construct a bioelectronic interface for protein‐induced neuromorphic interaction. As shown in **Figure** **3**a, an OECT is configured with a functionalized gate electrode, where target proteins bind to immobilized aptamers. This schematic highlights the architecture of the sensing platform and illustrates the localization of protein recognition at the gate interface.

Figure 3b conceptually depicts the biomolecular regulation of artificial synaptic behavior. The protein‐free state represents a non‐modulated interface (top), whereas biomolecule binding triggers ionic interactions and sustained electrical changes (bottom), enabling plasticity analogous to that observed in synaptic systems. This framework lays the foundation for translating biochemical cues into neuromorphic outputs, where protein‐regulated plasticity in artificial devices mirrors the way biological systems encode stimulus history, suggesting that such signals may carry perceptual value when paired with appropriate decoding mechanisms.^[^ ^36^ ^]^

IL‐6 recognition to emulate dynamic changes in signal transduction, demonstrating neuromorphic behavior analogous to synaptic systems. This includes transitions from short‐term plasticity (STP) to long‐term plasticity (LTP). It underscores its potential to mimic biochemical‐driven neural responses and generate signals with time‐dependent modulation.^[^ ^37^ ^]^ The response was characterized in PBS by measuring current changes (∆PSC) across IL‐6 concentrations ranging from 5 pm to 50 nm. As shown in Figure S13 (Supporting Information), the pulse‐induced current response increases with IL‐6 concentration, indicating concentration‐dependent signal modulation.

In pure electrolyte without targeted protein, applying 1‐second pulses (0.1–0.3 V, 0.1 V steps) results in transient postsynaptic current (PSC) responses that quickly return to baseline due to reversible ion migration (Figure 3c), consistent with STP. The relationship between ∆PSC and pulse amplitude is summarized in Figure 3d and Figure S14 (Supporting Information), showing current changes increasing from 18 to 97 µA. When electrolyte involve targeted protein molecules, as pulse voltage or IL‐6 concentration increases, current responses become more persistent, signaling a transition from STP to LTP.^[^ ^38^ ^]^ All neuromorphic measurements are repeated across multiple devices (*n* = 3), and the results show consistent synaptic behaviors with acceptable variability across batches. Similarly, increasing pulse duration (1–5 s, *V*_G_ = 0.1 V) under rising IL‐6 concentrations induces more sustained current changes (Figure 3e), reflecting enhanced memory effects consistent with synaptic strengthening (Figure 3f; Figure S15, supporting information). For instance, applying 10 successive pulses (*V*_G_ = 0.1 V, t = 1 s) leads to cumulative memory behavior, especially at higher IL‐6 levels (Figure 3g; Figure S16, Supporting information). In PBS alone, the current fully recovers after each pulse, preventing signal accumulation. In contrast, IL‐6 induces persistent baseline shifts and enhanced PSC amplitudes, evidencing a shift from transient to long‐lasting modulation.^[^ ^39^ ^]^ This memory retention is further amplified as the number of pulses increases from 5 to 15, accompanied by progressive de‐doping and reduced conductance (Figure 3h). To further confirm the biological relevance of these neuromorphic features, experiments are conducted using LPS‐treated THP‐1 cells, which naturally produce IL‐6. This device also shows current buildup and memory effects in response to repeated pulses, similar to the synaptic plasticity seen with direct IL‐6 stimulation. (Figure S17, Supporting Information).

To evaluate specificity, control biomolecules, including urea, lactic acid (LA), and fructose (Fru), are tested. As shown in Figure 3i and Figure S18 (Supporting information), only IL‐6 triggered significant cumulative effects under 10 pulse stimulation, while other biomolecules failed to induce long‐term plasticity or persistent conductance changes. Additional pulse width tests (1–5s) confirm the absence of memory effects from non‐specific biomolecules (Figure S19, supporting information), highlighting the device's selective responsiveness to IL‐6. These findings underscore the capability of the IL‐6‐responsive platform to mimic synaptic plasticity and selectively process protein‐based pathological cues, offering a foundation for future biohybrid interfaces that integrate molecular signals into adaptive neuromorphic architectures.

To fully understand how the device achieves this neuromorphic response and its integration into intelligent neural interfaces, it is essential to examine the working mechanism of the transistor, including its IL‐6 recognition process and signal modulation pathways. In the absence of IL‐6 (PBS only), ions are randomly distributed under equilibrium at *V*_G_ = 0 V (Figure S20a, supporting information). Upon applying a positive gate voltage (*V*_G_ > 0 V), an electric field drives cations toward the PEDOT: PSS channel, promoting cation doping and causing a reduction in hole concentration, which leads to a decrease in channel current. (Figure S20b, supporting information) Once the pulse ends, ions rapidly return to equilibrium, and the current quickly recovers, representing short‐term modulation in PBS (Figure S20c, supporting information). When IL‐6 is present, negatively charged IL‐6 molecules bind to the aptamer at the gate, attracting additional cations in their vicinity (Figure 3j). Upon pulsing (*V*_G_>0 V), these locally concentrated cations are further driven toward the channel, intensifying the de‐doping effect and causing a more pronounced current decrease (Figure 3k). After the pulse ends (*V*_G_ = 0 V), the excess cations diffuse back slowly, leading to a delayed recovery of the channel current (Figure 3l). This prolonged response reflects a memory behavior, mimicking synaptic long‐term modulation in the presence of IL‐6.^[^ ^40^ ^]^ This extended retention behavior arises from the IL‐6/aptamer complex modulating the local ionic distribution and dynamics at the gate–electrolyte interface. Specifically, the presence of IL‐6 alters the interfacial charge density, enhances local cation concentration, and slows ion relaxation, thereby facilitating more effective charge retention in the PEDOT:PSS channel. As a result, the device exhibits synaptic‐like plasticity, with a transition from transient to long‐term modulation driven by biological recognition.^[^ ^41^, ^42^ ^]^

### 2.4 IL‐6‐Induced Neuromorphic Plasticity in Serum

**Figure 4:**
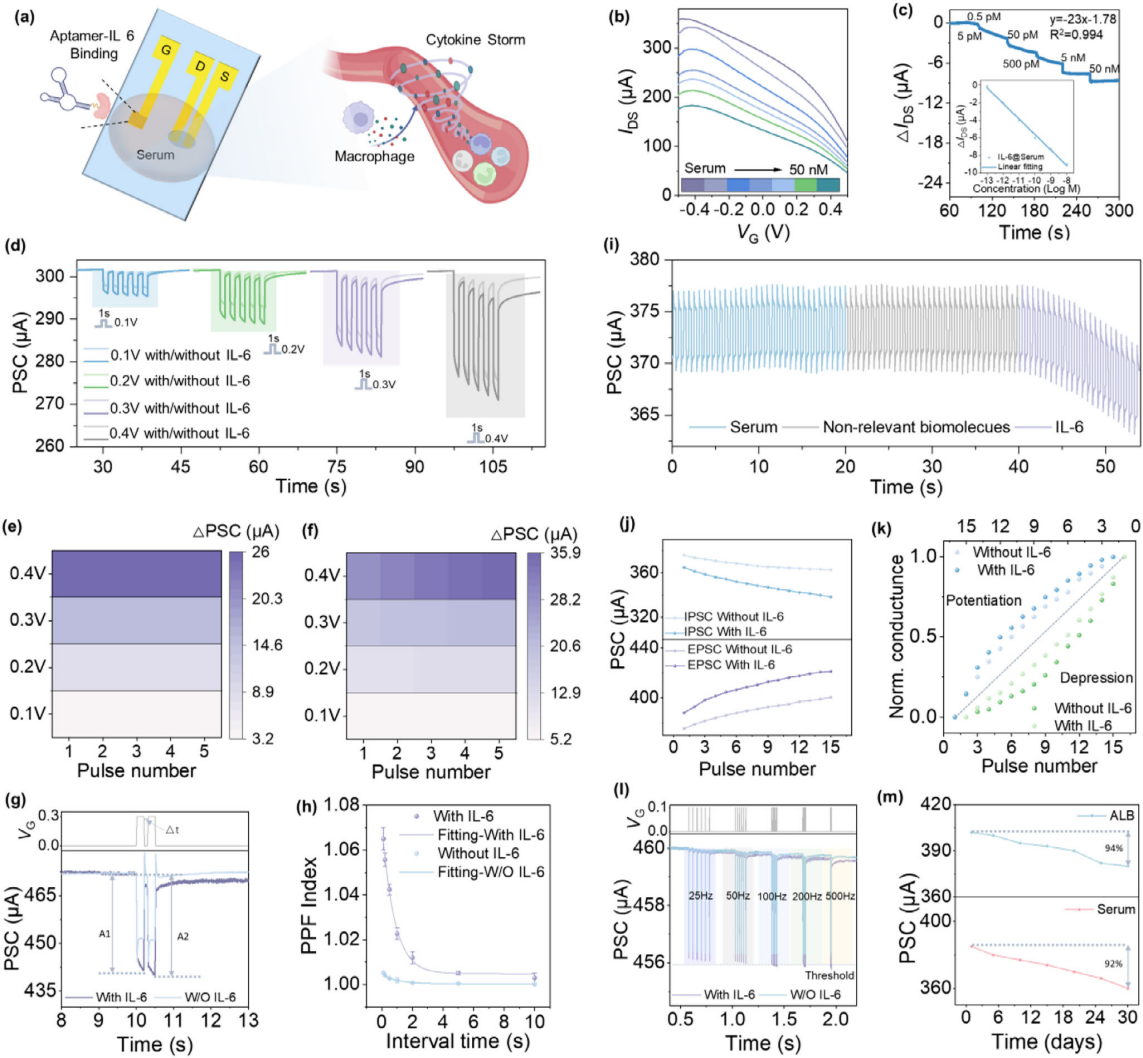
Functional and synaptic behaviors of the OECT‐based device in a serum environment simulating cytokine release syndrome (CRS). a)Schematic of the organic electrochemical device with protein–aptamer binding at the gate electrode in a serum‐based electrolyte and the blood vessel showing macrophages and cytokine release, simulating the CRS‐related inflammatory environment. b) Transfer characteristic curves of the device at the different IL‐6 concentrations in serum solution. c) Real‐time channel current response and the linear fit of Δ*I*_DS_ of the device at different IL‐6 concentrations in serum. d) Pulse‐dependent PSC responses with and without IL‐6 in serum. Heat map of spike current distribution in the serum e) without and f) with IL‐6. g) Potentiation triggered by a pair of presynaptic spikes h) Paired‐pulse facilitation (PPF) index and corresponding exponential fitting curves with and without IL‐6 with error bar. i) PSC responses of the device under voltage pulses in different serum solutions: pure serum solution (blue), serum solution containing non‐relevant biomolecules glucose (gray), and IL‐6 (purple), respectively. j) PSC modulation as a function of pulse number under positive and negative voltage stimuli. k) Comparison of weight change rate with/without IL‐6. l)PSC in response to stimulus trains of six 1‐ms stimulus spikes applied at different frequencies. m) Long‐term stability of PSC signals under single‐pulse stimulation in ALB and serum environments over 30 days.

Interleukin‐6 (IL‐6) is a key inflammatory cytokine involved in immune regulation, acute‐phase responses, and neuroimmune signaling.^[^ ^40^, ^43^, ^44^ ^]^ IL‐6 levels increase significantly in peripheral blood during infection or tissue injury, marking inflammatory stress.^[^ ^41^, ^42^ ^]^ Additionally, IL‐6 has emerged as a critical driver of cytokine release syndrome, a severe adverse effect observed in cancer immunotherapy.^[^ ^45^ ^]^Converting IL‐6 into neuromorphic electrical signals enables artificial systems to capture and represent such physiological states in a dynamic and structured form, supporting downstream neuromorphic processing under biologically relevant conditions. To evaluate this capability in physiologically complex environments, we employ human serum as the electrolyte and construct an inflammation‐mimicking model using IL‐6 (∼5 nm) at pathological concentrations. (**Figure** **4**a)

Transfer curves (Figure 4b) and real‐time current measurements (Figure 4c) demonstrate that increasing IL‐6 concentrations result in a current decrease through the PEDOT: PSS channel. The response exhibits strong linearity (y = –23 ×  –1.78, R^2^ = 0.994, where x = log [IL‐6]), confirming the device's high sensitivity and stable signal conversion in serum.

As shown in Figure 4d, the PSC values at different pulse amplitudes, ranging from 0.1 to 0.4 V, exhibit clear differences in both serum with/without IL‐6. In the absence of IL‐6, the device shows minimal current changes under pulse stimulation, with the heatmap displaying a uniform distribution (Figure 4e). This behavior reflects a baseline state of the artificial synaptic device, where chemical signals exert negligible influence on neuromorphic behavior. In contrast, the presence of IL‐6 induces pronounced memory effects under repeated stimulation, revealing the dynamic modulation of neuromorphic plasticity mediated by protein interaction. Specifically, after each pulse, the current amplitude initially increases but gradually stabilizes with successive pulses, demonstrating a transition from short‐term to long‐term neuromorphic behavior. The heatmap (Figure 4f) highlights these transitions as non‐uniform distributions, demonstrating the capability of the device to translate protein molecular interactions into distinctive electrical patterns.

To evaluate the synaptic gain and tunability, we apply two successive pulses (*V*_G_ = 0.3 V) with varying pulse intervals (Δt) ranging from 0.1 to 10 s to study the paired‐pulse facilitation (PPF) effect. The PPF index, defined as the ratio of the second PSC peak (A2) to the first peak (A1​), reflects the dynamic memory effect in the system (Figure 4g). As shown in Figure 4h, the PPF index exhibits a clear dependence on the presence of IL‐6 in the serum environment. Under IL‐6 conditions, the PPF index reaches a maximum facilitation of 106% at Δt = 0.1s and gradually declines to 100.05% as Δt increases to 10 s, indicating a sustained synaptic enhancement effect. In contrast, without IL‐6, the PPF index exhibits a narrower tunable range, from 100.5% to 100.01%, suggesting a weaker facilitation effect. To quantify the dynamic changes, the decay of the PPF index was fitted using a double exponential function:−

(1) $$\mathrm{PPF}\;\mathrm{index}=C_{1}\mathrm{Exp}\;\left(-\Delta t/\tau 1\right)+C_{2}\mathrm{Exp}\left(-\Delta t/\tau 2\right)+C_{0}$$

C_0_ represents the asymptotic PPF index, C_1_ and C_2_ are the weight factors for the two facilitation components, and τ1 and τ2 are the characteristic relaxation times associated with fast and slow ionic relaxation, respectively. For the IL‐6 condition, the extracted time constants were τ1 = 0.60s and τ2 = 3.10s. In contrast, in the absence of IL‐6, the device exhibits negligible memory effects, and the signal decays too rapidly to extract corresponding τ values through reliable exponential fitting. The prolonged τ under IL‐6 exposure reflects enhanced retention behavior, likely resulting from IL‐6‐mediated modulation of charge carrier dynamics and electrolyte interactions. This supports the role of IL‐6 in promoting long‐term signal persistence, analogous to synaptic plasticity in biological systems. The selectivity of IL‐6‐induced synaptic plasticity in complex media is achieved by applying voltage pulses to the gate electrode in serum. As shown in Figure 4i, no cumulative memory effect is observed under successive voltage pulses when using either pure serum or serum containing non‐specific biomolecules such as glucose (5 nm). In contrast, the introduction of IL‐6 (5 nm) led to a significant increase in channel current accumulation over repeated pulses, indicating a specific, protein‐driven modulation of the synaptic response.

As shown in Figure 4j and Figure S21 (Supporting information), the device exhibits cumulative memory behavior under repeated ±0.3 V pulse stimulation, with a progressive increase in PSC responses observed in the presence of IL‐6, indicating enhanced synaptic modulation compared to the condition without IL‐6. The PSC responses under continuous pulse stimulation reflect the evolution of synaptic weight in the neuromorphic interface in Figure 4k. Figure 4k further illustrates synaptic weight evolution. Without IL‐6, normalized conductance changes follow a near‐linear trend, suggesting limited modulation. In contrast, IL‐6 induces increasing deviation from the diagonal, forming a curved trajectory that reflects nonlinear potentiation and depression. This behavior implies a history‐dependent memory encoding process, highlighting IL‐6's role in enhancing synaptic adaptability within the neuromorphic interface.

To explore frequency‐dependent behavior, PSC amplitudes were recorded under spike trains ranging from 25 to 500 Hz (Figure 4l). Without IL‐6, the PSC remained above a fixed threshold across all frequencies, indicating a low‐pass filtering effect. In contrast, the IL‐6 group exceeded the threshold only at high frequencies (>100 Hz), indicating frequency selectivity and its frequency‐dependent filtering function. Long‐term stability is evaluated in serum and albumin over 30 days. Figure 4m shows that the device retained 94% and 92% of its initial spike response in albumin and serum, respectively, underscoring excellent signal retention in biologically complex media. Together, these findings demonstrate that the device not only functions reliably in complex biological media but also translates protein‐level molecular recognition into neuromorphic dynamics. This molecular‐to‐electrical translation, enhanced by IL‐6 interaction, paves the way for bioelectronic interfaces that bridge immune signals with adaptive neural‐like computation, forming the basis for autonomous sensing in bio‐integrated systems.

### 2.5 In Vivo Synaptic Behaviors of Neuromorphic Modulation and Classification

**Figure 5:**
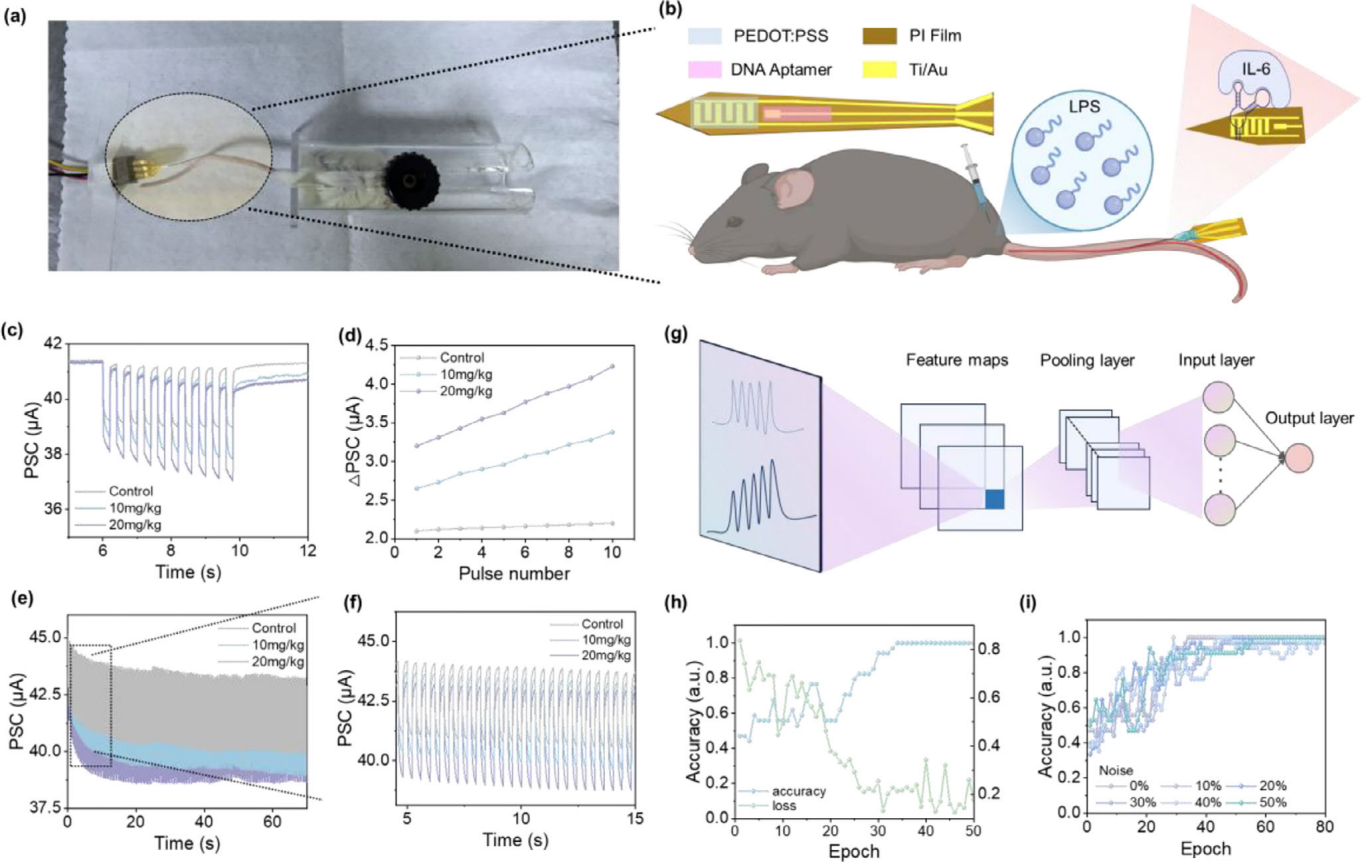
In Vivo synaptic communication induced by the implantable AAAST device. a) Photograph of the in vivo experimental setup, where the flexible AAAST device is implanted into the tail vein of a mouse via an indwelling needle. b) Schematic illustration of the implantation process, IL‐6 recognition through aptamer‐protein binding on the device surface, and the structure of the flexible, implantable device. c) PSC responses of the implanted device under 10 voltage pulses in different in vivo blood environments. The gray line represents the response without LPS injection (control), the blue line indicates the response after 10 mg kg^−1^ LPS injection, and the purple line corresponds to 20 mg kg^−1^ LPS. Enhanced PSC amplitude is observed with increasing LPS concentration, indicating strengthened neuromorphic behavior under inflammatory conditions. d) ΔPSC values of the device under different in vivo conditions, reflecting concentration‐dependent signal enhancement. e) PSC response of the implanted device to multiple consecutive voltage pulses. f) Local magnification of the PSC response, highlighting dynamic signal characteristics. g) Schematic diagram of the artificial neural network (ANN) structure used for signal analysis. h) Training curves of the accuracy and loss of the ANN. i) Classification accuracy of the ANN under various input noise intensities, demonstrating signal robustness.

Building upon the in vitro demonstration of protein‐regulated synaptic behavior, we further validated the device's neuromorphic functionality under physiologically relevant conditions. A miniaturized, flexible OECT was fabricated on a 200 µm‐wide polyimide substrate (Figure S22, supporting information) and implanted into the tail vein of live mice using an indwelling needle, enabling minimally invasive, real‐time biochemical signal transduction in vivo. (**Figure** **5**a,b)

Flow cytometry was performed on peripheral blood leukocytes to confirm humanization as shown in Figure S23 (Supporting information). Cells were stained with mCD45‐FITC and hCD45‐APC to distinguish between mouse‐ and human‐derived leukocytes. Among singlet live cells, 47.58% were mCD45⁺/hCD45− (mouse leukocytes) and 52.41% were mCD45−/hCD45⁺ (human leukocytes), indicating successful engraftment of human immune cells capable of producing human IL‐6. To assess real‐time neuromorphic performance under physiological conditions, a humanized mouse model of LPS‐induced sepsis was established via intraperitoneal injection of 10 or 20 mg kg^−1^ LPS, with a control group injected with an equal volume of PBS. ELISA analysis confirmed successful model induction, showing a dose‐dependent increase in mouse serum IL‐6 levels (Figure S24, Supporting Information).

Following implantation of the flexible OECT device into the tail vein, the neuromorphic electrical responses were recorded under 10 consecutive voltage pulses. As shown in Figure 5c, the device exhibits enhanced neuromorphic behavior in LPS‐treated mice, with greater signal persistence and cumulative effects compared to controls. The amplitude of current change increases progressively with LPS dose, as quantified in Figure 5d. Furthermore, with a higher number of consecutive pulses, the devices show pronounced long‐term modulation effects (Figure 5e,f), consistent with biological long‐term plasticity.

To evaluate the potential for real‐time disease state recognition, we develop a machine learning classification model based on the ResNet‐18 architecture, which is trained using RGB images converted from electrical response data collected under both normal and LPS‐induced inflammatory conditions (Figure 5g). These images capture the temporal and spatial features of the signal patterns, enabling the model to distinguish between physiological and pathological states. A detailed schematic is provided in Figure S25 (Supporting Information). As shown in Figure 5h, the accuracy plot shows the model effectively distinguished between healthy and pathological states. After 30 training epochs, the classification accuracy reached 100%. Meanwhile, the loss plot illustrates a steady decrease in the error across the training process, confirming that the model is effectively minimizing its classification error. Even under artificial noise interference, the model maintained robust performance, with classification accuracy remaining effective after 50% noise levels (Figure 5i), indicating strong noise tolerance. These results suggest that the neuromorphic signals generated by the device encode physiologically relevant information that can be interpreted by neural network models, supporting their potential role in the autonomous perception of biochemical states. This closed‐loop process from molecular recognition to neuromorphic encoding to computational interpretation lays a conceptual and technical foundation for future bioelectronic interfaces capable of perceptual integration in biohybrid systems.

## 3 Conclusion

In conclusion, we have demonstrated an OECT‐based artificial synapse in which the synaptic weight is selectively modulated by an aptamer in response to IL‐6 molecules under gate voltage pulses. The aptamer‐functionalized gate enables highly specific molecular recognition, translating biochemical inputs into adaptive neuromorphic outputs that emulate key synaptic features, including plasticity transitions and long‐term depression. The device exhibits excellent sensitivity and selectivity toward IL‐6 across physiologically relevant media, including PBS, albumin, and human serum, validating its robust biochemical‐to‐electrical signal conversion. Notably, protein‐induced transitions from linear to nonlinear synaptic behavior, paired‐pulse facilitation, and frequency‐dependent filtering are observed, highlighting the system's capability to encode biochemical states into dynamic, memory‐effect electrical signatures. Furthermore, under in vivo conditions, the device maintains stable operation after implantation into the bloodstream of mice, demonstrating that the device enables seamless communication and functional integration with biological systems. Coupled with signal classification based on ANN, the system successfully distinguishes between normal and pathological states, representing a significant step toward autonomous molecular perception.

This device holds great promise as an artificial neuron for sensing, memory, and processing biochemical signals both in vitro and in vivo, providing a crucial foundation for the development of intelligent bio‐interfaces that bridge biological systems with next‐generation diagnostics, personalized health monitoring, and advanced human‐machine interaction.

## 4 Experimental Section

### Materials

Poly(3,4‐ethylenedioxythiophene):polystyrene sulfonate (PEDOT:PSS) aqueous solution, phosphate‐buffered saline (PBS, 1×, pH 7.4), and albumin (ALB) were procured from Sigma–Aldrich and were being kept at 4 °C for storage. Potassium hexacyanoferrate(III) [K_3_Fe(CN)_6_], potassium hexacyanoferrate(II)trihydrate [K_4_Fe(CN)_6_·3H_2_O], mercaptohexanol (MCH), dodecylbenzenesulfonic acid, and (3‐glycidyloxypropyl)trimethoxysilane are all sourced from Shanghai Adamas Reagent Co., Ltd. Interleukin‐6 (IL‐6) was obtained from Novoprotein Scientific Inc. The IL‐6 aptamer sequence, C_6_HS‐SH5’GGTGGCAGGAGGACTATTTATTTGCTTTTCT3’, was synthesized and purified by Sangong Biotech (Shanghai, China).

### Fabrication of OECT

Patterned Cr/Au source, drain, and gate electrodes were meticulously deposited onto glass substrates using a shadow mask technique and thermal evaporation. An initial thin layer of chromium (≈5 nm in thickness) served as an adhesion layer, ensuring the secure bonding of the subsequent gold layer (≈40 nm thick). To prepare the PEDOT:PSS mixture for the channel, 6 vol.% ethylene glycol, 0.1 vol.% dodecylbenzene sulfonic acid, and 1 vol.% (3‐glycidyloxypropyl)trimethoxysilane are added to the PEDOT:PSS aqueous solution. The PI substrate pre‐patterned with Ti/Au electrodes was treated with O_2_ plasma for 5 min (Harrick PDC‐32G‐2, 18 W). Subsequently, a thin PEDOT:PSS film is formed between the source and drain electrodes on the substrate by spin‐coating the PEDOT:PSS mixture at 1500 rpm for 30 s, followed by thermal annealing at 120 °C for 10 min.

### Ethics Statement

All animal experimental procedures in this study were reviewed and approved by the Animal Ethics Committee of the Experimental Animal Center of Tongji University (approval number: TJBH17125103). This study strictly adhered to the requirements of the National Institutes of Health's “Guide for the Care and Use of Laboratory Animals” (8th edition) and the ARRIVE 2.0 guidelines. The “3Rs principle” (Replacement, Reduction, Refinement) was strictly implemented to ensure animal welfare. The experimental animals were housed in a specific pathogen‐free (SPF) environment with controlled environmental parameters: temperature (22±1°C), humidity (50% ± 10%), and a 12‐h light‐dark cycle. They had free access to standard laboratory chow and filtered drinking water. Analgesic measures and humane endpoint protocols were applied when necessary to minimize animal suffering to the greatest extent.

### Statistical Analysis

All data shown in this manuscript are representative. Each experiment was repeated at least three times to ensure reproducibility. Error bars represent standard deviations unless otherwise noted.

## Conflict of Interest

The authors declare no conflict of interest.

## Supporting information



Supporting Information

## Data Availability

The data that support the findings of this study are available in the supplementary material of this article.

## References

[1] P. Gkoupidenis , Y. Zhang , H. Kleemann , H. Ling , F. Santoro , S. Fabiano , A. Salleo , Y. van de Burgt , Nat. Rev. Mater. 2024, 9, 134.

[2] K. He , C. Wang , Y. He , J. Su , X. Chen , Chem. Rev. 2023, 123, 13796. 37976052 10.1021/acs.chemrev.3c00527

[3] S. Chen , T. Zhang , S. Tappertzhofen , Y. Yang , I. Valov , Adv. Mater. 2023, 35, 2301924. 10.1002/adma.202301924 37199224

[4] M. Zhang , Z. Tang , X. Liu , J. Van der Spiegel , Nat. Electron. 2020, 3, 191.

[5] M. J. Devine , J. T. Kittler , Nat. Rev. Neurosci. 2018, 19, 63. 10.1038/nrn.2017.170 29348666

[6] T. Wang , M. Wang , J. Wang , L. Yang , X. Ren , G. Song , S. Chen , Y. Yuan , R. Liu , L. Pan , Z. Li , W. R. Leow , Y. Luo , S. Ji , Z. Cui , K. He , F. Zhang , F. Lv , Y. Tian , K. Cai , B. Yang , J. Niu , H. Zou , S. Liu , G. Xu , X. Fan , B. Hu , X. J. Loh , L. Wang , X. Chen , Nat. Electron. 2022, 5, 586.

[7] X. Xu , H. Zhang , L. Shao , R. Ma , M. Guo , Y. Liu , Y. Zhao , Angew. Chem., Int. Ed. 2023, 62, 202302723. 10.1002/anie.202302723 37178394

[8] S. T. Keene , C. Lubrano , S. Kazemzadeh , A. Melianas , Y. Tuchman , G. Polino , P. Scognamiglio , L. Cinà , A. Salleo , Y. van de Burgt , F. Santoro , Nat. Mater. 2020, 19, 969. 32541935 10.1038/s41563-020-0703-y

[9] X. Liu , D. Wang , W. Chen , Y. Kang , S. Fang , Y. Luo , D. Luo , H. Yu , H. Zhang , K. Liang , L. Fu , B. S. Ooi , S. Liu , H. Sun , Nat. Commun. 2024, 15, 7671. 39227588 10.1038/s41467-024-51194-z PMC11371922

[10] M. Schiller , T. L. Ben‐Shaanan , A. Rolls , Nat. Rev. Immunol. 2021, 21, 20. 32811994 10.1038/s41577-020-0387-1

[11] F. Zipp , S. Bittner , D. P. Schafer , Immunity 2023, 56, 914. 37163992 10.1016/j.immuni.2023.04.011 PMC10233069

[12] D. Kim , J.‐S. Lee , Adv. Funct. Mater. 2022, 32, 2200497.

[13] S. A. Jones , B. J. Jenkins , Nat. Rev. Immunol. 2018, 18, 773. 30254251 10.1038/s41577-018-0066-7

[14] L. Fayad , M. J. Keating , J. M. Reuben , S. O'Brien , B.‐N. Lee , S. Lerner , R. Kurzrock , Blood 2001, 97, 256. 11133769 10.1182/blood.v97.1.256

[15] D. L. Gruol , Neuropharmacology 2015, 96, 42. 25445486 10.1016/j.neuropharm.2014.10.023 PMC4446251

[16] Y. Gao , D. T. Nguyen , T. Yeo , S. B. Lim , W. X. Tan , L. E. Madden , L. Jin , J. Y. K. Long , F. A. B. Aloweni , Y. J. A. Liew , M. L. L. Tan , S. Y. Ang , S. D. O. Maniya , I. Abdelwahab , K. P. Loh , C.‐H. Chen , D. L. Becker , D. Leavesley , J. S. Ho , C. T. Lim , Sci. Adv. 7, abg9614. 10.1126/sciadv.abg9614 PMC8139589 34020961

[17] C. Diacci , B. Burtscher , M. Berto , T.‐P. Ruoko , S. Lienemann , P. Greco , M. Berggren , M. Borsari , D. T. Simon , C. A. Bortolotti , F. Biscarini , ACS Appl. Mater. Interfaces 2024, 16, 61467. 38141020 10.1021/acsami.3c12397 PMC11565573

[18] S. Li , K. Huang , Q. Fan , S. Yang , T. Shen , T. Mei , J. Wang , X. Wang , G. Chang , J. Li , Biosens. Bioelectron. 2019, 136, 91. 31039492 10.1016/j.bios.2019.04.034

[19] B. Xiao , J. Li , S. Guo , Y. Zhang , M. Peng , H. Yu , M. Deng , J. Wang , L. Yu , X. Wang , ACS Appl. Mater. Interfaces 2022, 14, 1626. 34968026 10.1021/acsami.1c21706

[20] F. Mariani , F. Decataldo , F. Bonafè , M. Tessarolo , T. Cramer , I. Gualandi , B. Fraboni , E. Scavetta , ACS Appl. Mater. Interfaces 2024, 16, 61446. 37966461 10.1021/acsami.3c10576 PMC11565569

[21] E. Macchia , K. Manoli , B. Holzer , C. Di Franco , M. Ghittorelli , F. Torricelli , D. Alberga , G. F. Mangiatordi , G. Palazzo , G. Scamarcio , L. Torsi , Nat. Commun. 2018, 9, 3223. 30104563 10.1038/s41467-018-05235-z PMC6089965

[22] K. Guo , S. Wustoni , A. Koklu , E. Díaz‐Galicia , M. Moser , A. Hama , A. A. Alqahtani , A. N. Ahmad , F. S. Alhamlan , M. Shuaib , A. Pain , I. McCulloch , S. T. Arold , R. Grünberg , S. Inal , Nat. Biomed. Eng. 2021, 5, 666. 34031558 10.1038/s41551-021-00734-9

[23] L. Vanzetti , L. Pasquardini , C. Potrich , V. Vaghi , E. Battista , F. Causa , C. Pederzolli , Surf. Interface Anal. 2016, 48, 611.

[24] H. Yu , H. Zhang , J. Li , Z. Zhao , M. Deng , Z. Ren , Z. Li , C. Xue , M. G. Li , Z. Chen , ACS Sens. 2022, 7, 3923. 36472865 10.1021/acssensors.2c01990

[25] B. Wang , C. Zhao , Z. Wang , K.‐A. Yang , X. Cheng , W. Liu , W. Yu , S. Lin , Y. Zhao , K. M. Cheung , H. Lin , H. Hojaiji , P. S. Weiss , M. N. Stojanović , A. J. Tomiyama , A. M. Andrews , S. Emaminejad , Sci. Adv. 8, abk0967. 10.1126/sciadv.abk0967 PMC8730602 34985954

[26] A. Koklu , S. Wustoni , K. Guo , R. Silva , L. Salvigni , A. Hama , E. Diaz‐Galicia , M. Moser , A. Marks , I. McCulloch , R. Grünberg , S. T. Arold , S. Inal , Adv. Mater. 2022, 34, 2202972. 10.1002/adma.202202972 35772173

[27] J. Tan , B. Hao , Z. Liu , F. Bai , R. Yang , H. Hao , BIO Web Conf. 2017, 8.

[28] K. L. Rhinehardt , V. S. A. , M. R. V. , S. Marinella , G. Srinivas , J. Biomol. Struct. Dyn. 2018, 36, 1934. 28592206 10.1080/07391102.2017.1338619

[29] A. Shimabukuro‐Vornhagen , P. Gödel , M. Subklewe , et al., J. ImmunoTher. Cancer 2018, 6, 56. 29907163 10.1186/s40425-018-0343-9 PMC6003181

[30] J. Y. Hansen , G. Shafiei , R. D. Markello , K. Smart , S. M. L. Cox , M. Nørgaard , V. Beliveau , Y. Wu , J.‐D. Gallezot , É. Aumont , S. Servaes , S. G. Scala , J. M. DuBois , G. Wainstein , G. Bezgin , T. Funck , T. W. Schmitz , R. N. Spreng , M. Galovic , M. J. Koepp , J. S. Duncan , J. P. Coles , T. D. Fryer , F. I. Aigbirhio , C. J. McGinnity , A. Hammers , J.‐P. Soucy , S. Baillet , S. Guimond , J. Hietala , et al., Nat. Neurosci. 2022, 25, 1569. 36303070 10.1038/s41593-022-01186-3 PMC9630096

[31] C. Huang , Y. Wang , X. Li , L. Ren , J. Zhao , Y. Hu , L. Zhang , G. Fan , J. Xu , X. Gu , Z. Cheng , T. Yu , J. Xia , Y. Wei , W. Wu , X. Xie , W. Yin , H. Li , M. Liu , Y. Xiao , H. Gao , L. Guo , J. Xie , G. Wang , R. Jiang , Z. Gao , Q. Jin , J. Wang , B. Cao , Lancet 2020, 395, 497. 31986264 10.1016/S0140-6736(20)30183-5 PMC7159299

[32] M. Deng , Z. Ren , H. Zhang , Z. Li , C. Xue , J. Wang , D. Zhang , H. Yang , X. Wang , J. L. Deng , Z. Ren , H. Zhang , Z. Li , C. Xue , J. Wang , D. Zhang , H. Yang , X. Wang , J. Li , Adv. Sci. 2023, 10, 2205886. 10.1002/advs.202205886 PMC9896035 36480308

[33] H. Liu , J. Song , Z. Zhao , S. Zhao , Z. Tian , F. Yan , Adv. Sci. 2024, 11, 2305347. 10.1002/advs.202305347 PMC11251571 38263718

[34] M. Deng , H. Yang , H. Zhang , C. Li , J. Chen , W. Tang , X. Wang , Z. Chen , J. Li , Adv. Healthcare Mater. 2024, 13, 2302117. 10.1002/adhm.202302117 37922499

[35] S. Kang , T. Kishimoto , Exp. Mol. Med. 2021, 53, 1116. 34253862 10.1038/s12276-021-00649-0 PMC8273570

[36] W. Liu , Z. Du , Z. Duan , L. Li , G. Shen , Nat. Commun. 2024, 15, 5635. 38965218 10.1038/s41467-024-49907-5 PMC11224243

[37] H. Shim , F. Ershad , S. Patel , Y. Zhang , B. Wang , Z. Chen , T. J. Marks , A. Facchetti , C. Yu , Nat. Electron. 2022, 5, 660.

[38] Y. Zang , H. Shen , D. Huang , C.‐A. Di , D. Zhu , Adv. Mater. 2017, 29, 1606088. 10.1002/adma.201606088 28225213

[39] A. Citri , R. C. Malenka , Neuropsychopharmacology 2008, 33, 18. 17728696 10.1038/sj.npp.1301559

[40] T. Xiong , C. Li , X. He , B. Xie , J. Zong , Y. Jiang , W. Ma , F. Wu , J. Fei , P. Yu , L. Mao , Science 2023, 379, 156. 36634194 10.1126/science.adc9150

[41] C. Ausilio , C. Lubrano , D. Rana , G. M. Matrone , U. Bruno , F. Santoro , Adv. Sci. 2024, 11, 2305860 10.1002/advs.202305860 PMC11251551 38702931

[42] C. Lubrano , U. Bruno , C. Ausilio , F. Santoro , Adv. Mater. 2022, 34, 2110194. 10.1002/adma.202110194 35174916

[43] S. Kumari , R. Dhapola , D. H. Reddy , Apoptosis 2023, 28, 943. 37186274 10.1007/s10495-023-01848-y

[44] S. K. Beura , R. Dhapola , A. R. Panigrahi , P. Yadav , R. Kumar , D. H. Reddy , S. K. Singh , Med. Res. Rev. 2023, 43, 1835. 37132460 10.1002/med.21965

[45] E. C. Morris , S. S. Neelapu , T. Giavridis , M. Sadelain , Nat. Rev. Immunol. 2022, 22, 85. 34002066 10.1038/s41577-021-00547-6 PMC8127450

