# Supplementary material for: Aptamer‐Mediated Artificial Synapses for Neuromorphic Modulation of Inflammatory Signaling via Organic Electrochemical Transistor
Source: Adv Sci (Weinh). 2025 Aug 4;12(40):e09545. doi: 10.1002/advs.202509545 (PMC12561412; doi:10.1002/advs.202509545)
Supplement: Supplementary file 1 — Supporting Information [file ADVS-12-e09545-s001.docx]

Supporting Information

Aptamer-Mediated Artificial Synapses for Neuromorphic Modulation of Inflammatory Signaling via Organic Electrochemical Transistor

*Yuqing Ding, You Kuai, Rongpei Li, Xinzhao Xu, Bo Wang, Zhihui Wang, Yanfang Liu, Yuchao Dong, Shunjie Chen^*^, Meng Guo^*^, Yunqi Liu, Yan Zhao^*^*

**Experiment Section**

*Materials:* Poly(3,4-ethylenedioxythiophene):polystyrene sulfonate (PEDOT:PSS) aqueous solution, phosphate-buffered saline (PBS, 1X, pH 7.4), and albumin (ALB) were procured from Sigma-Aldrich and are being kept at 4°C for storage. Potassium hexacyanoferrate(III) [K_3_Fe(CN)_6_], potassium hexacyanoferrate(II)trihydrate [K_4_Fe(CN)_6_·3H_2_O], mercaptohexanol (MCH), dodecylbenzenesulfonic acid, and (3-glycidyloxypropyl)trimethoxysilane were all sourced from Shanghai Adamas Reagent Co., Ltd. Interleukin-6 (IL-6) was obtained from Novoprotein Scientific Inc. The IL-6 aptamer sequence, C_6_HS-SH5’GGTGGCAGGAGGACTATTTATTTGCTTTTCT3’, was synthesized and purified by Sangong Biotech (Shanghai, China).

*Fabrication of OECT:* Patterned Cr/Au source, drain, and gate electrodes were meticulously deposited onto glass substrates using a shadow mask technique and thermal evaporation. An initial thin layer of chromium (approximately 5 nm in thickness) served as an adhesion layer, ensuring the secure bonding of the subsequent gold layer (approximately 40 nm thick). To prepare the PEDOT:PSS mixture for the channel, 6 vol.% ethylene glycol, 0.1 vol.% dodecylbenzene sulfonic acid and 1 vol.% (3-glycidyloxypropyl)trimethoxysilane are added to the PEDOT:PSS aqueous solution. The PI substrate pre-patterned with Ti/Au electrodes was treated with O_2_ plasma for 5min (Harrick PDC-32G-2, 18 W). Subsequently, a thin PEDOT:PSS film is formed between the source and drain electrodes on the substrate by spin-coating the PEDOT:PSS mixture at 1500 rpm for 30s, followed by thermal annealing at 120 °C for 10 minutes.

*Functionalization of the Au gate with aptamers:* To functionalize the gate electrodes with DNA aptamers, the thiolated aptamer (0.5uM) was reduced by tris(2-carboxyethyl) phosphine (TCEP) at room temperature for 60 minutes. Subsequently, 10 μL of the aptamer solution was drop cast on the surface of the gate electrode and incubated for 60 minutes, followed by drying with nitrogen gas. To prevent non-specific adsorption, the surface was subsequently blocked with mercaptohexanol (MCH), involving a 1h incubation with a 3 mM MCH solution at a temperature of 4°C.

*Fabrication of micro-sized implantable electrodes:* Two layers of photoresist (sacrificial layer LOR 3A and photoresist S1813) were spin-coated on a PI substrate sequentially, followed by baking at 150 °C for 5 min and 110 °C for 1 min on a hotplate, respectively. Subsequently, photolithography was performed using ultraviolet lithography (Microwriter ML3, Durham Magneto Optics Ltd.) as designed and Ti/Au (5/40 nm) were deposited through the thermal evaporator (Angstrom Engineering). A lift-off process by immersing the above electrodes into the remover PG stripper solution for 30 min and acetone for 30 min was carried out to define the source, drain, and gate electrodes.

*Preparation of cells:* The human monocytic leukemia cell line (THP-1) was purchased from the American Type Culture Collection (ATCC, Manassas, VA, USA). THP-1 cells were cultured in RPMI 1640 medium (BasalMedia, L210KJ) supplemented with 10% fetal bovine serum (FBS; Gibco, Waltham, MA, USA) and 1% penicillin/streptomycin (P/S; Biosharp, Hefei, China) at 37°C under 5% CO₂. For stimulation, 3×10⁶ THP-1 cells were seeded in 10 cm culture dishes and treated with 5 μg/mL LPS.

*Humanized Mouse Models:* Humanized mice (6-8 weeks old, 20-22 g) were generated by transplanting human umbilical cord blood-derived CD34⁺ hematopoietic stem cells into immunodeficient mice expressing human IL-3 and GM-CSF, promoting multilineage immune cell reconstitution and enabling human IL-6 secretion. Mice were purchased from Vital River Laboratory Animal Technology Co., Ltd. (Beijing, China) and housed under specific pathogen-free (SPF) conditions at 23 °C with a 12-hour light/dark cycle and free access to food and water. All procedures were approved by the Scientific Research Committee of Naval Medical University (Shanghai, China). For sepsis modeling, mice were anesthetized with 3% isoflurane (maintained at 2%) and injected intraperitoneally with 0, 10, or 20 mg/kg LPS (serotype O111:B4, Sigma-Aldrich). Four hours later, an organic synaptic device was intravenously implanted via the tail vein using a 26G needle.

*Enzyme-Linked Immunosorbent Assay (ELISA):* L-6 levels in mouse serum and cell culture supernatants were measured using commercial ELISA kits (Dakewe, Beijing, China) according to the manufacturer’s instructions.

*Surface plasmon resonance (SPR) measurements:* IL-6 protein was immobilized on the chip via EDC/NHS chemistry, and a reference channel was prepared in parallel without protein. The running buffer was PBS-P+ (pH 7.4) containing 5% DMSO, with solvent correction performed using a DMSO calibration curve. Aptamer solutions were injected at increasing concentrations (30 μL/min, 60 s), and regeneration was carried out with 10 mM glycine-HCl (pH 2.0). Kinetic parameters were obtained by global fitting to a 1:1 Langmuir model using Biacore Insight software.

*Artificial Neural Network (ANN):* RGB images used for ANN training are generated from electrical response data obtained under both normal and LPS-stimulated conditions. Each image encodes the temporal and spatial patterns of current changes and was resized to 768 × 512 pixels. Prior to training, images were normalized using the mean and standard deviation values from the CIFAR-100 dataset. Data augmentation techniques, including random cropping (to 700 × 480 pixels), horizontal flipping, and rotation, were applied to enhance generalization. The dataset consisted of 10 experimental images for training and 10 for testing, with non-overlapping sample sets defined by train.txt and test.txt. Model training used stochastic gradient descent (SGD) with momentum (0.9), weight decay (1e-4), and a multi-step learning rate scheduler with warm-up. Cross-entropy loss was used as the loss function. Gradient noise was artificially introduced during training to simulate biological signal variability and improve model robustness. Performance metrics (loss and accuracy) were monitored using TensorBoard.

*Characterization and measurement:* The surface morphology of the semiconductor films was examined using atomic force microscopy (AFM) on a Bruker Dimension ICON, operated in tapping mode, and scanning electron microscopy (SEM) with a ThermoFisher Scientific instrument, both at ambient temperature. Elemental composition analysis was conducted using X-ray photoelectron spectroscopy (XPS) on a Thermo Fisher Nexsa system, employing a monochromatic Al Kα radiation source.

The electrochemical properties of the gold electrode, both before and following aptamer functionalization, were evaluated through electrochemical impedance spectroscopy (EIS) and cyclic voltammetry (CV) in a three-electrode configuration with an electrochemical workstation. A platinum wire served as the counter electrode, while an Ag/AgCl electrode functioned as the reference electrode. The gold electrode was designated as the working electrode. All electrochemical measurements were performed in a phosphate-buffered saline (PBS) solution at pH 7.4, containing 5 mM Fe(CN)_6_^3−^/Fe(CN)_6_^4−^. For cyclic voltammetry, the potential window spanned from −0.5 to 0.8 V, with a scan rate of 50 mV s^−1^. Electrochemical impedance spectra were acquired with an AC amplitude of 5 mV, covering a frequency range of 0.1 to 100 kHz.

Device characterization was conducted using a Keysight B2902, with all measurements taken under a constant drain voltage (*V*_DS_) of 0.1 V. The electrical characteristics of the device are recorded by measuring transfer characteristics (drain current *I*_DS_ as a function of gate voltage *V*_G_) and current-time response (*I*_D_ as a function of time). For the measurements of synaptic behavior of the device under voltage pulses, a constant DC voltage was applied onto the drain electrode (V_ds_ = 0.1 V), and the source electrode was grounded, while programmed voltage pulses were applied onto the gate electrode.


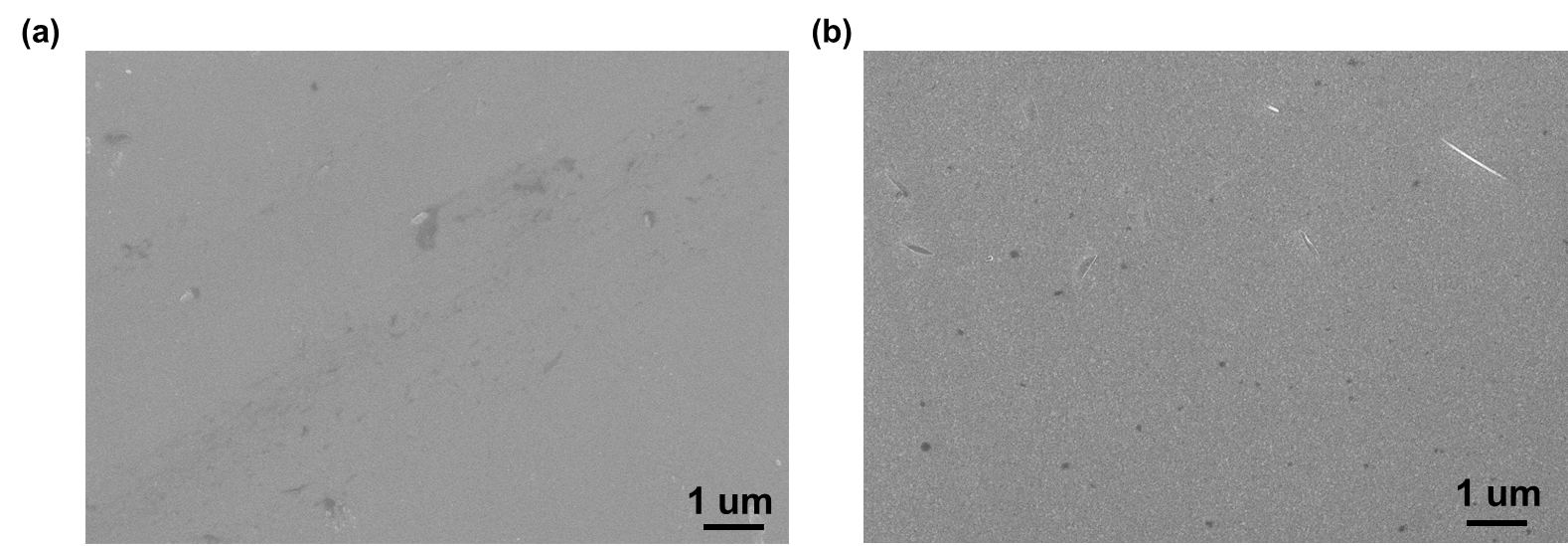


Figure S1 SEM image for the Au gate (a) without and (b) with aptamer function


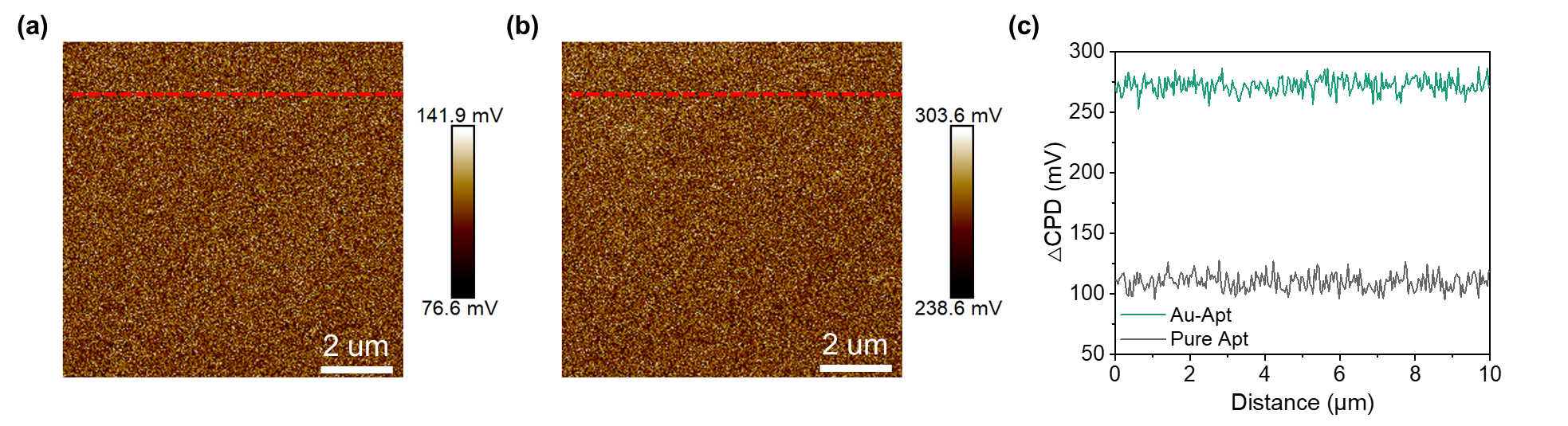


Figure S2 KPFM image for the Au gate (a) without, (b)with aptamer function, and (c)corresponding surface potential


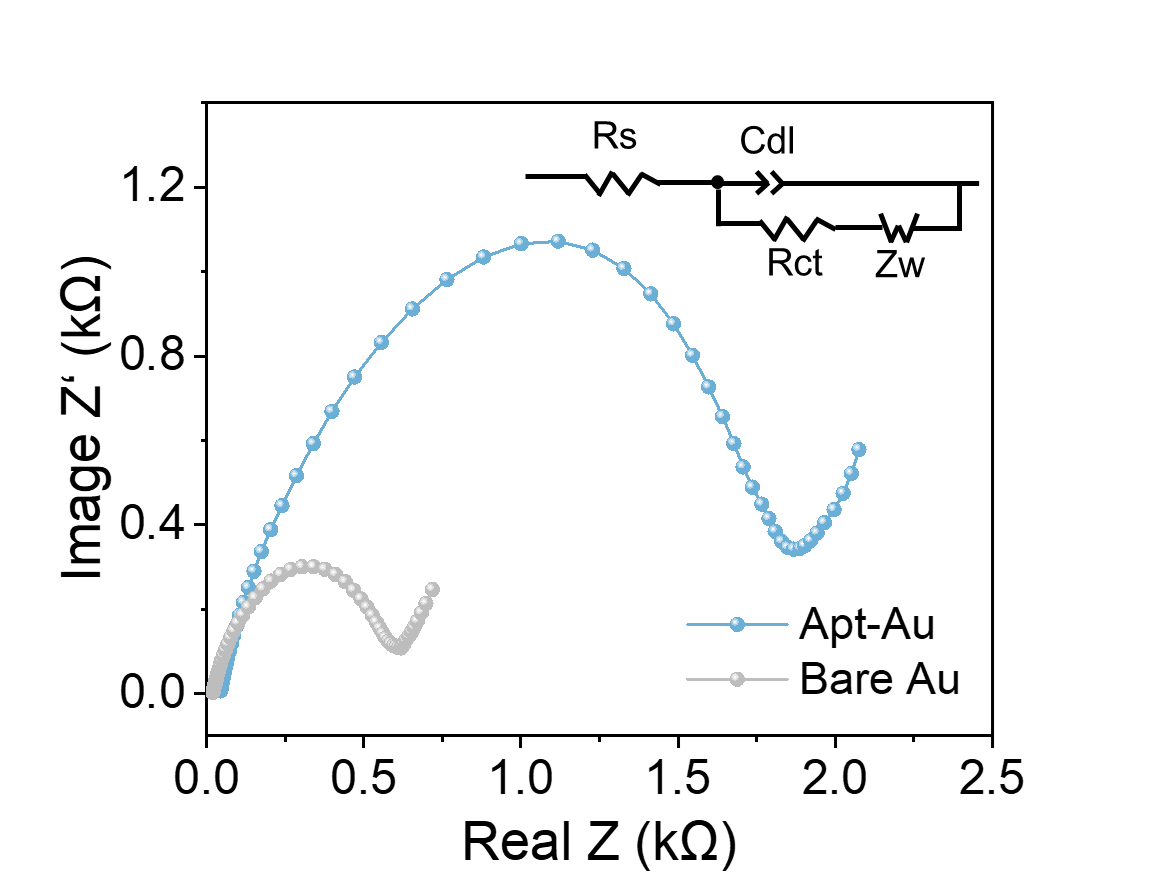


Figure S3 Nyquist plot of the Au electrode before and after aptamer functionalization


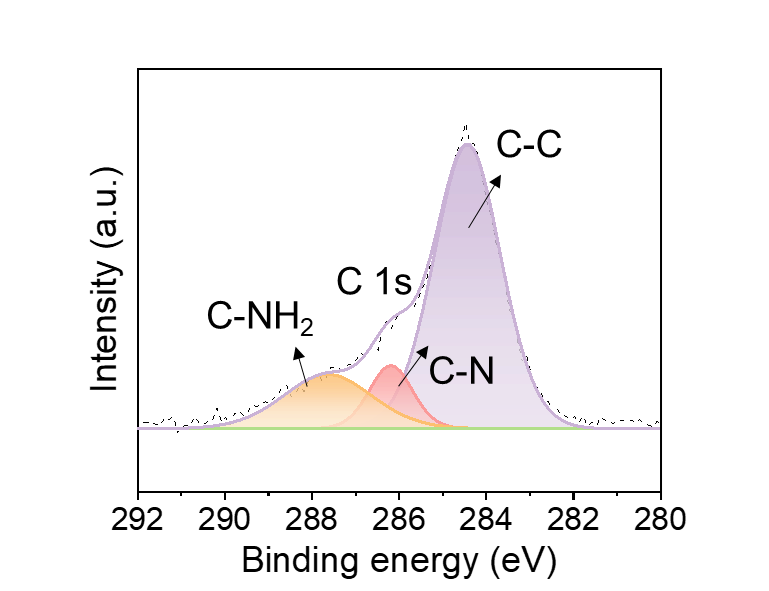


Figure S4 High-resolution C 1 s for aptamer functionalized Au gate.

Figure S5 Surface plasmon resonance (SPR) analysis for IL-6 protein and its aptamer

Figure S6 Comparison of current responses at varying aptamer concentrations (0.2 μM, 0.5 μM, and 1 μM).


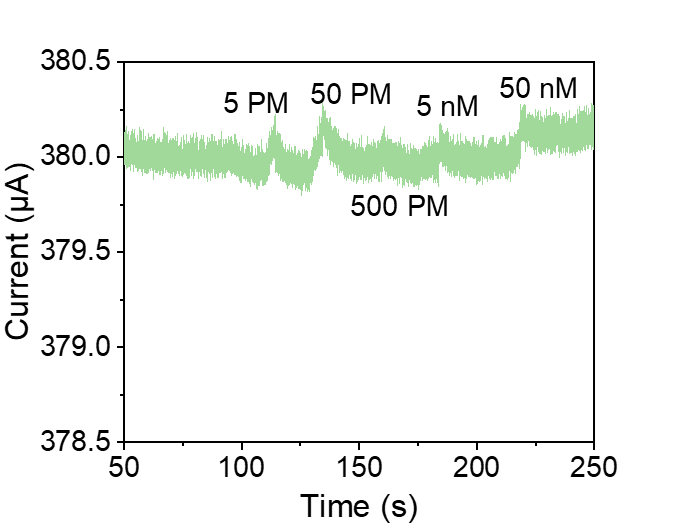


Figure S7 Real-time channel current response curves of the device without aptamer functionalization under varying IL-6 concentrations.


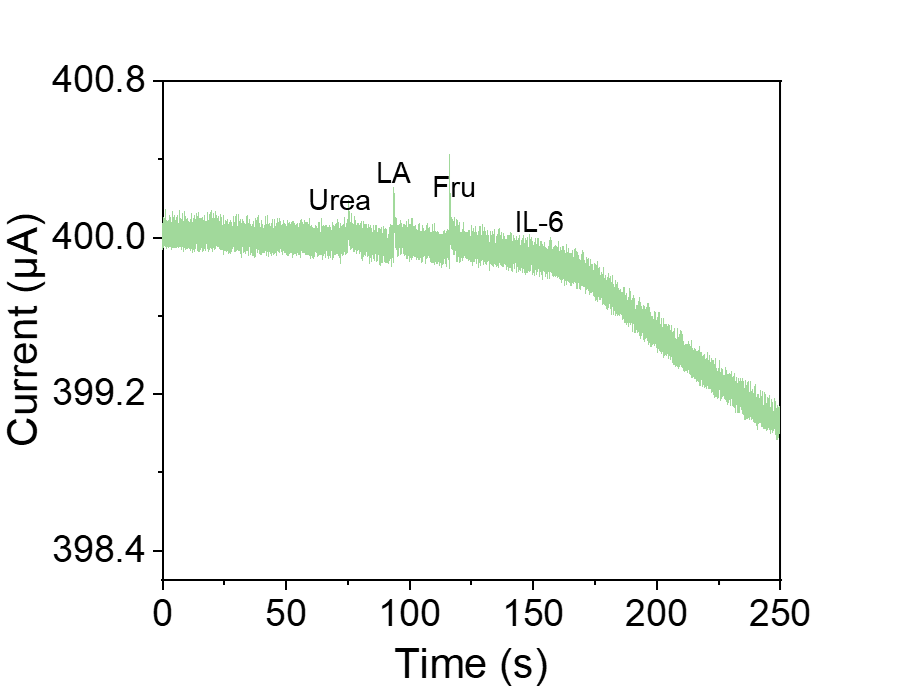


Figure S8 Real-time channel current responses of the device upon exposure to non-relevant molecules and IL-6.


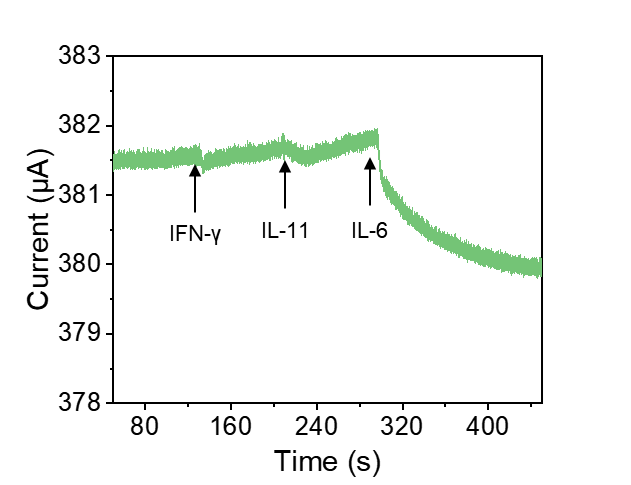


Figure S9 Real-time channel current responses of the device upon exposure to other cytokines and IL-6.


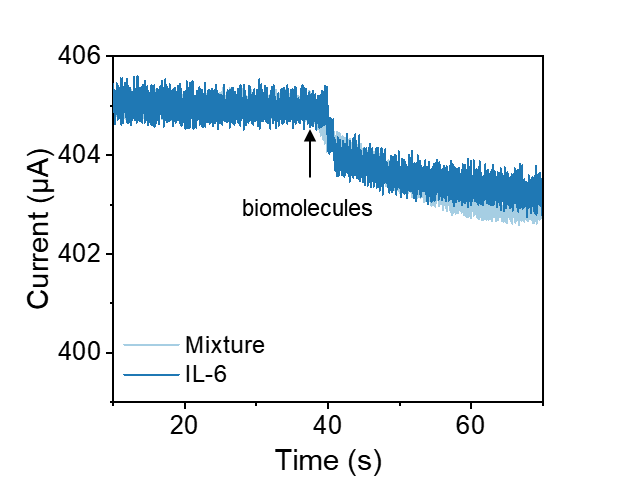


Figure S10 Real-time channel current responses of the device upon exposure to the mixture of cytokines and IL-6.

Figure S11 Capacitance-frequency curve for the device before and after IL-6 binding


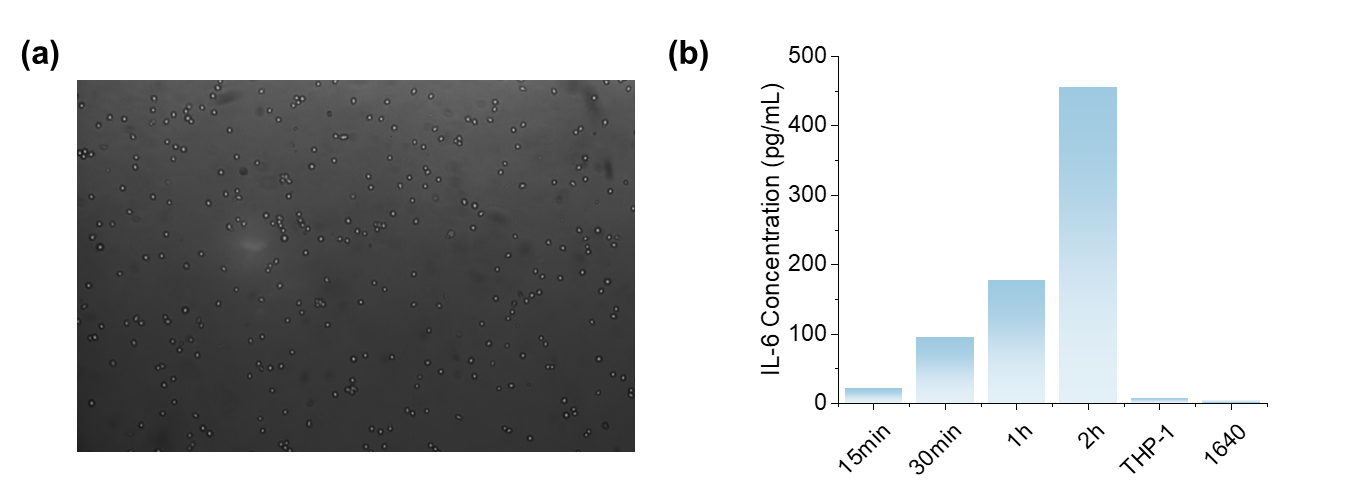


Figure S12 (a) Optical image of THP-1 cells. (b) ELISA measurement of IL-6 concentrations in LPS-stimulated THP-1 cells (15 min to 2 h), compared with control groups (untreated THP-1 cells and 1640 medium).


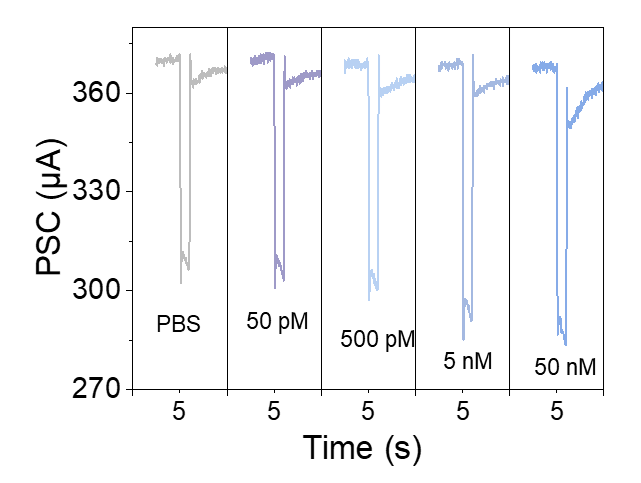


Figure S13 PSC responses of the device at varying IL-6 concentrations (50 pM to 50 nM) under *V*_G_ = 0.3 V and *V*_DS_ = 0.1 V.

Figure S14 ∆PSC of the device in PBS solution at varied IL-6 concentrations with different pulse voltage.

Figure S15 ∆PSC of the device in PBS solution at varied IL-6 concentrations with different pulse duration.

Figure S16 △PSC of the device in PBS solution at varied IL-6 concentrations with different pulse numbers.

Figure S17 Cumulative pulse stimulations applied to the device in 1640 medium, THP-1 cells, and THP-1 cells treated with LPS.

Figure S18 ΔPSC responses of the device in PBS solutions containing different biomolecules (urea, LA, Fru, and IL-6) under repeated pulse voltage.


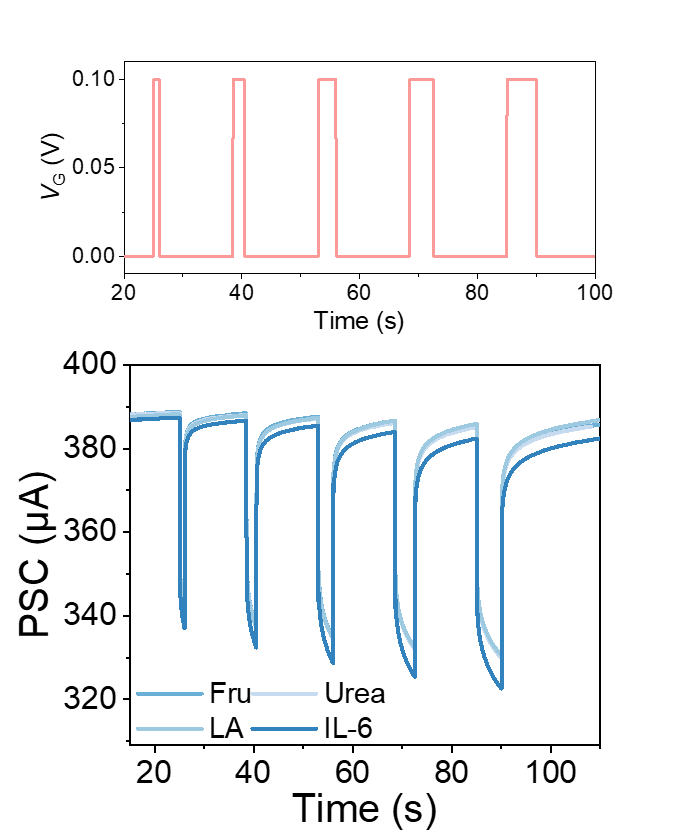


Figure S19 PSC responses of the device in PBS solutions containing different biomolecules (urea, LA, fru, and IL-6) under varying pulse widths.


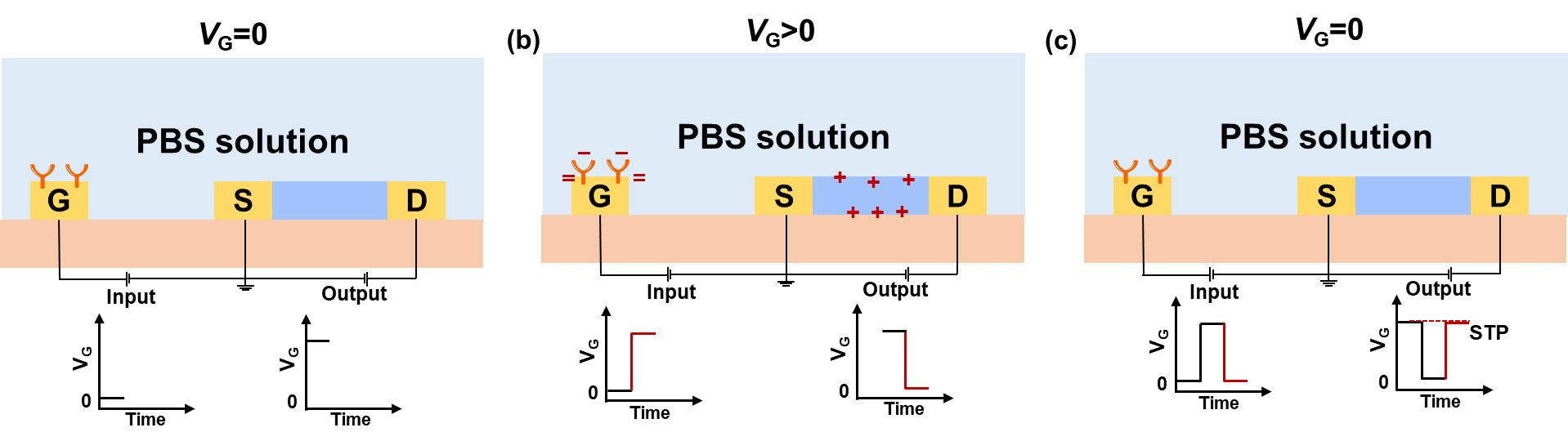


Figure S20 Schematic illustration of the working principle of the device without IL-6 when (a)no voltage pulse is applied, (b) the channel current is induced by the applied voltage pulse, and (l) when the pulse is removed.


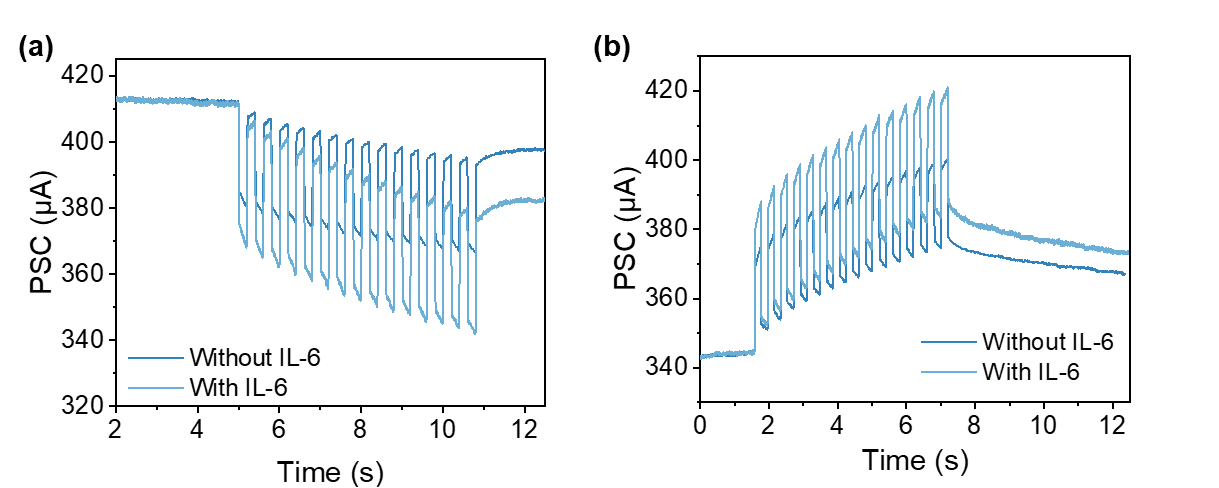


Figure S21 PSC responses demonstrating the cumulative memory behavior of the device: (a) depression under repeated 0.3V pulse voltages; (b) potentiation under repeated −0.3V pulse voltages.


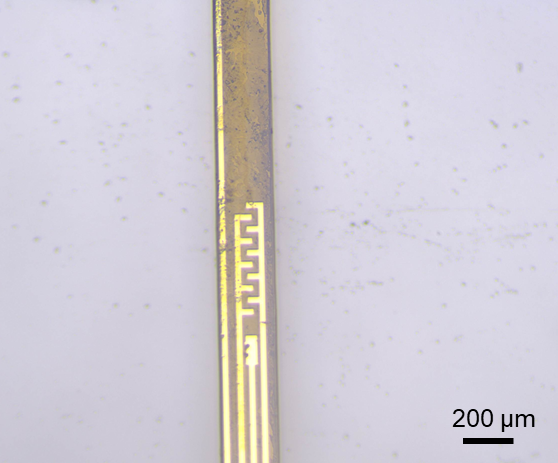


Figure S22 The optical image of the implantable device


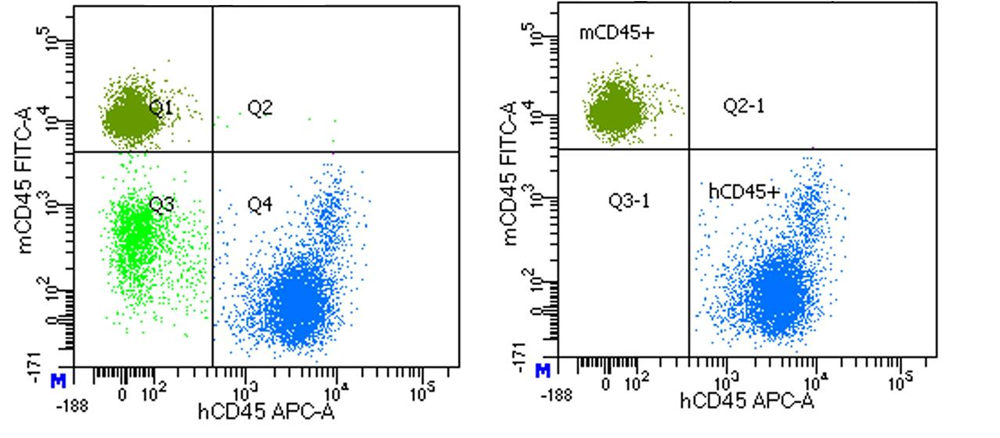


Figure S23 Flow cytometry validation of humanized mice.

Figure S24 ELISA measurement of IL-6 concentrations in mice stimulated with different concentrations of LPS.


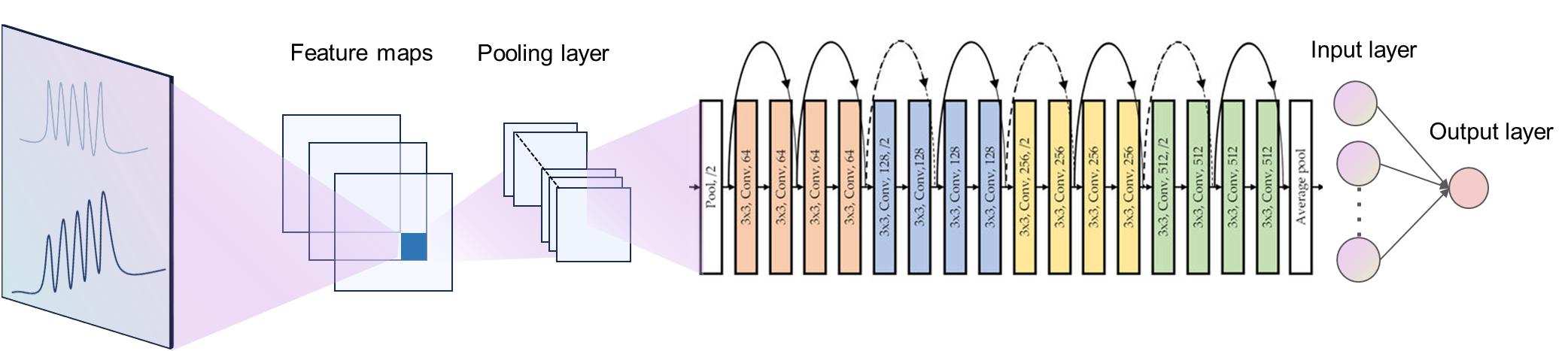


Figure S25 Schematic illustration of the ANN architecture.
